# Facial Emotion Recognition via Fusion of Deep and Handcrafted Features

**DOI:** 10.3390/s26144522

**Published:** 2026-07-16

**Authors:** Seo Eun Cha, Beom Kwon

**Affiliations:** 1 Division of Information Science, Dongduk Women’s University, Seoul 02784, Republic of Korea; seoun9100@gmail.com; 2 Division of Interdisciplinary Studies in Cultural Intelligence, Dongduk Women’s University, Seoul 02748, Republic of Korea

**Keywords:** convolutional neural network, deep feature, facial emotion recognition, facial landmarks, feature fusion, handcrafted features, histogram of oriented gradients

## Abstract

Facial emotion recognition plays an important role in affective computing and human–computer interaction. Although convolutional neural network (CNN)-based methods have demonstrated remarkable performance, deep features alone may not sufficiently capture subtle geometric deformations and local texture variations, particularly under limited training data and challenging real-world conditions. To address this limitation, this study proposes a hybrid framework that integrates CNN-based deep features with handcrafted geometric and texture features. Specifically, 17 landmark-based angular features extracted from the eyebrows, eyes, nose, and mouth are combined with histogram of oriented gradients (HOG) features extracted from the nose and mouth regions through feature-level concatenation. The proposed method was extensively evaluated on three controlled datasets (JAFFE, CK+, and KDEF) and two large-scale in-the-wild datasets (RAF-DB and AffectNet). Five-fold cross-validation, leave-one-subject-out cross-validation, statistical significance analysis using paired *t*-tests, computational efficiency analysis, and comparisons with conventional handcrafted methods, standard CNN models, transfer learning-based methods, and recent hybrid feature-fusion methods were performed to comprehensively validate the proposed approach. Experimental results demonstrated consistent improvements across different datasets, evaluation protocols, and CNN backbone networks while maintaining a favorable balance between recognition performance and computational efficiency. These findings demonstrate that handcrafted geometric and local texture features effectively complement CNN-based deep representations, providing a robust and generalizable framework for facial emotion recognition across both controlled and large-scale in-the-wild datasets.

## 1. Introduction

Emotion recognition is a fundamental component of human communication because it enables individuals to perceive and interpret the emotional states of others. The ability to perceive emotions accurately is essential for effective social interaction and decision-making. Beyond interpersonal communication, emotion recognition has become increasingly important in various professional domains such as customer service, healthcare, and intelligent systems. In these fields, recognizing users’ emotional states and responding appropriately can significantly enhance the effectiveness of services and support [1].

Among the various modalities used for emotion recognition, facial emotion recognition has attracted considerable attention because of its practicality and non-intrusive nature in real-world environments [2]. In healthcare applications, facial emotion recognition systems can be used to monitor patients and assess their level of discomfort or pain [3]. In the automotive industry, facial emotion recognition enables the detection of driver fatigue or stress by analyzing facial expressions. When signs of fatigue are detected, warning signals can be provided to the driver to prevent potential accidents [4]. In the marketing industry, facial emotion recognition is used to analyze consumers’ emotional responses during product evaluation and shopping experiences, enabling companies to collect valuable feedback on their products and services [5].

Facial emotion recognition is also a key research topic in affective computing. Affective computing refers to the study of computational systems that can recognize, interpret, and model human emotional states using various modalities such as facial expressions, speech signals, and physiological responses [6]. By incorporating emotion-aware capabilities into computing systems, affective computing aims to improve the quality of human–computer interaction (HCI). These technologies have been applied to various domains, including intelligent devices, remote learning, digital entertainment, social robots, and intelligent toys, providing more personalized and adaptive user experiences [7].

Various approaches have been proposed for emotion recognition, including methods based on speech signals [8], electroencephalography (EEG) [9], and physiological sensors [10]. However, many of these approaches require specialized equipment, which limits their practical applicability in everyday environments. In contrast, facial emotion recognition provides a more intuitive and efficient solution because emotional expressions can be captured directly from facial images using widely available cameras. Consequently, facial emotion recognition has become one of the most widely studied topics in affective computing and HCI [11].

Recent advances in deep learning have significantly improved the performance of facial emotion recognition systems. Convolutional neural networks (CNNs) are widely used for extracting deep features from facial images and have demonstrated strong performance in facial analysis and emotion recognition tasks [12]. Nevertheless, deep learning-based approaches sometimes fail to capture subtle geometric variations in facial expressions, particularly when the training dataset is limited or imbalanced. Therefore, integrating complementary handcrafted features with deep representations has recently attracted increasing attention in facial emotion recognition research. In particular, combining deep features with handcrafted features that explicitly encode geometric and local texture characteristics of facial expressions has shown promising performance by providing complementary information that cannot be fully captured by deep representations alone [13].

Motivated by these observations, this paper proposes a hybrid facial emotion recognition framework that integrates CNN-based deep features with handcrafted geometric and texture features derived from facial landmarks. To comprehensively validate the proposed approach, extensive experiments were conducted using multiple evaluation protocols and benchmark datasets, including controlled laboratory datasets, subject-independent evaluation, and large-scale in-the-wild datasets. The main contributions of this paper are summarized as follows:

- A hybrid facial emotion recognition framework is proposed by combining CNN-based deep features with handcrafted geometric and texture features.
- A set of handcrafted features consisting of 17 landmark-based angular features and nose/mouth-region histogram of oriented gradients (HOG) features is designed to capture complementary geometric and local texture information associated with facial expressions.
- Extensive experiments were conducted on three controlled datasets (JAFFE, CK+, and KDEF) together with two large-scale in-the-wild datasets (RAF-DB and AffectNet) under multiple evaluation protocols.
- Comprehensive experimental validation, including five-fold cross-validation, leave-one-subject-out (LOSO) cross-validation, statistical significance analysis, computational efficiency evaluation, and comparisons with representative benchmark methods, demonstrates that the proposed feature-fusion strategy consistently improves facial emotion recognition performance across diverse datasets and evaluation scenarios.

The remainder of this paper is organized as follows: Section 2 reviews related work on hand-crafted feature-based, deep learning-based, and hybrid facial emotion recognition methods. Section 3 describes the proposed facial emotion recognition framework, including handcrafted feature extraction, CNN-based deep feature extraction, and feature-level fusion. Section 4 presents the experimental setup, including the datasets, benchmark methods, implementation details, and evaluation protocols. Section 5 reports the experimental results obtained under five-fold cross-validation, while Section 6 presents the subject-independent evaluation results obtained using LOSO cross-validation. Section 7 and Section 8 report the experimental results on the official RAF-DB and AffectNet train/test splits, respectively. Section 9 discusses the experimental findings and limitations of the proposed method. Finally, Section 10 concludes the paper and suggests directions for future research.

## 2. Related Work

### 2.1. Handcrafted Feature-Based Facial Emotion Recognition

Early studies on facial emotion recognition primarily relied on handcrafted features extracted from facial images. These approaches aim to represent facial expressions using manually designed descriptors that capture texture or geometric information from facial regions. Based on these characteristics, handcrafted feature-based methods can be broadly categorized into texture-based approaches and geometric feature-based approaches.

Texture-based methods focus on capturing local appearance variations in facial images. For example, the authors of [14] proposed a facial expression recognition method using Gabor filter-based features, which effectively capture spatial frequency and orientation information. Similarly, the authors of [15] introduced the compound local binary pattern (CLBP) to overcome the limitations of the traditional local binary pattern (LBP). Unlike LBP, which encodes only the sign of intensity differences, CLBP incorporates both the sign and magnitude, resulting in improved recognition performance.

On the other hand, geometric feature-based approaches utilize the spatial relationships between facial components. The authors of [16] combined HOG features with landmark-based geometric features to capture both appearance and structural information. Their results showed that combining multiple feature types leads to better performance than using individual features. Similarly, the authors of [17] integrated edge-based features obtained using Canny edge detection with HOG descriptors, demonstrating improved performance across multiple datasets.

In addition to individual feature extraction methods, several studies have explored hybrid handcrafted feature approaches that combine texture-based and geometric features to enhance representation capability. In particular, combining appearance-based features, such as HOG or LBP, with geometric features derived from facial landmarks enables the representation of both local texture variations and structural relationships of facial components. Such complementary feature representations have been shown to significantly improve facial emotion recognition performance [18].

Despite their effectiveness, handcrafted feature-based methods have inherent limitations. Since these features are manually designed, they may not fully capture the complex and subtle variations in facial expressions. As a result, their performance is often constrained by the representational capacity of the selected features.

### 2.2. Deep Learning-Based Facial Emotion Recognition

With the rapid advancement of deep learning techniques, CNNs have become the dominant approach for facial emotion recognition. Unlike traditional handcrafted feature-based methods, CNN-based approaches automatically learn hierarchical feature representations from facial images, enabling more robust and discriminative modeling of facial expressions. Based on their architectural design and training strategies, deep learning-based facial emotion recognition methods can be broadly categorized into CNN architectures trained from scratch, transfer learning-based approaches, and data augmentation-based approaches.

Early deep learning-based facial emotion recognition studies focused on training CNNs from scratch using task-specific architectures. For instance, the author of [19] proposed a deep CNN model for facial expression recognition using the FER-2013 dataset, demonstrating that CNNs can effectively learn discriminative features directly from raw pixel data. Similarly, the authors of [20] introduced a deep neural network architecture for facial emotion recognition, achieving competitive performance by training the model end-to-end on large-scale datasets. These studies established the effectiveness of CNN-based feature learning without relying on pre-trained models.

To further improve performance, many studies have adopted transfer learning techniques, which leverage pre-trained models on large-scale datasets. The authors of [21] utilized a pre-trained VGG network as an initialization model and fine-tuned the CNN architecture for facial emotion recognition tasks. By transferring knowledge from large-scale image datasets, the model achieved improved performance compared to training from scratch. Similarly, the authors of [22] fine-tuned a VGG-16 model pre-trained on the ImageNet dataset using the FER-2013 dataset. Their study systematically analyzed the effects of training configurations, including epochs, optimizers, batch normalization, and data balancing strategies, demonstrating that deep CNN models can outperform traditional methods when properly optimized.

In addition, data augmentation-based approaches have also been widely explored to address the limitations of small and imbalanced facial emotion recognition datasets. For example, the author of [23] proposed a CNN-based method based on the LeNet-5 architecture using the JAFFE dataset. To mitigate the problem of limited training data, a convolutional autoencoder (CAE) was employed to generate additional training samples through interpolation in the latent space. The experimental results demonstrated that the proposed data augmentation strategy significantly improved recognition accuracy.

In addition to standard CNN architectures, more advanced deep learning models have been explored in recent studies. For instance, deeper architectures such as ResNet [24] and DenseNet [25] have been applied to facial emotion recognition to capture more complex feature representations. Furthermore, attention mechanisms have been introduced to focus on discriminative facial regions, improving recognition performance by emphasizing important features related to emotional expressions [26]. These approaches highlight the growing trend toward leveraging deeper and more sophisticated architectures for improved facial emotion recognition performance.

Despite the success of deep learning-based methods, several challenges remain. CNN-based approaches often require large-scale annotated datasets to achieve optimal performance. However, many facial emotion recognition datasets are relatively small and imbalanced, which can lead to overfitting and reduced generalization capability. In addition, deep features may not fully capture subtle geometric variations in facial expressions, particularly when fine-grained structural information is required. These limitations have motivated recent research efforts to combine deep features with complementary handcrafted features, which can explicitly represent geometric or structural characteristics of facial expressions, thereby improving recognition performance.

### 2.3. Hybrid Facial Emotion Recognition Approaches

To overcome the limitations of both handcrafted feature-based methods and deep learning-based approaches, recent studies have explored hybrid facial emotion recognition methods that combine deep features with handcrafted features [27]. These methods aim to leverage the complementary strengths of both feature types to improve recognition performance.

Handcrafted features are effective in explicitly representing geometric structures and local texture patterns of facial expressions, while deep features extracted from CNNs can capture high-level semantic representations. By integrating these complementary features, hybrid approaches can enhance both the discriminative power and robustness of facial emotion recognition systems.

Several studies have demonstrated the effectiveness of hybrid feature representations. For example, prior work has combined local texture descriptors, such as LBP or HOG, with geometric features derived from facial landmarks to capture both appearance and structural information [28]. Other studies have further integrated these handcrafted features with deep features extracted from CNN models, showing that such combinations can improve recognition performance compared to using either feature type alone.

In addition, some hybrid approaches have focused on multi-level feature fusion strategies [29]. These methods combine low-level handcrafted features with high-level deep features at different stages of the network, enabling the model to capture both fine-grained local variations and global facial patterns. Experimental results from these studies indicate that hybrid feature fusion can significantly improve recognition accuracy, particularly in scenarios with limited training data [30].

Despite these advantages, existing hybrid approaches still face several challenges [31]. In many cases, the selection of handcrafted features is not systematically optimized, and the fusion strategies are often heuristic or task-specific. Furthermore, effectively balancing the contributions of deep features and handcrafted features remains a challenging problem.

In this context, this study investigates a simple feature combination strategy and demonstrates that combining deep features with handcrafted features consistently improves recognition performance compared to using each feature independently. By leveraging complementary characteristics of both feature types, the proposed approach captures both high-level semantic representations and detailed geometric information of facial expressions.

## 3. Proposed Method

### 3.1. Overall Framework

**Figure 1:**
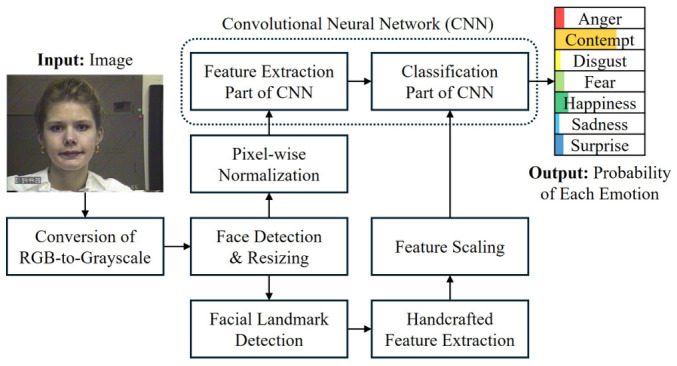
Overall framework of the proposed facial emotion recognition method.

Figure 1 illustrates the overall framework of the proposed facial emotion recognition method. As shown in the figure, the proposed system receives an RGB facial image as input. The RGB image contains three channels that represent color information such as skin tone, illumination, and saturation. However, these color-related factors are not directly related to emotional expression and may introduce unnecessary variability during the recognition process. Therefore, the input RGB image is first converted into a grayscale image with a single channel. This preprocessing step reduces computational complexity while preserving the structural information of facial expressions.

After preprocessing, the facial region is detected from the input image and resized to a fixed resolution for consistent processing. The resized facial image is then normalized and provided as the input to the CNN model for deep feature extraction. In parallel, facial landmarks are detected from the facial image to extract handcrafted features representing geometric and local structural characteristics of facial expressions. The extracted handcrafted features are further scaled before being combined with the deep features obtained from the CNN model. Finally, the fused feature representation is used for emotion classification, producing the probability distribution for each emotion class.

### 3.2. Face Detection and Image Preprocessing

**Figure 2:**
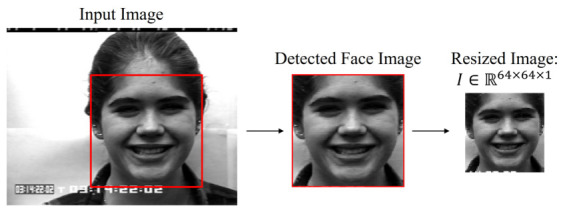
Face detection and resizing process of the input image.

Facial images often contain background regions that are unrelated to emotional expressions, and such irrelevant information may negatively affect recognition performance. Therefore, as shown in Figure 2, the proposed method first detects the facial region from the input image to remove unnecessary background information.

In this study, the get_frontal_face_detector() function provided by the Dlib library is used for face detection. This detector returns a rectangular bounding box corresponding to the detected frontal face region in the image. After face detection, the facial region is cropped from the original image.

Since the detected face sizes vary depending on the original image resolution and face scale, it is necessary to normalize the input size before feeding the images into the CNN. Therefore, all detected face images are resized to a fixed resolution of 64×64 pixels. This resizing process provides a consistent input representation and enables stable feature extraction during CNN training. For convenience, the resized grayscale image is denoted as $I\in R^{64\times 64\times 1}$, where the last dimension represents a single-channel intensity value.

### 3.3. Landmark-Based Handcrafted Feature Extraction

**Figure 3:**
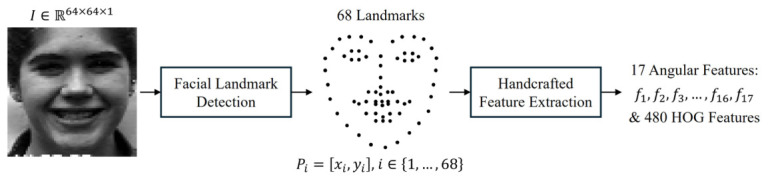
Overview of landmark-based handcrafted feature extraction.

The proposed method extracts handcrafted features from facial regions to complement the deep features extracted by the CNN model. These handcrafted features are designed to represent structural and local appearance variations that are closely related to emotional expressions. As illustrated in Figure 3, the proposed handcrafted features consist of landmark-based angular features and HOG features.

To extract geometric information from facial components, facial landmarks are first detected using the Dlib shape predictor, which identifies 68 landmark points on the face. Let i∈{1,…,68} denote the index of each landmark point, and the coordinate of the *i*-th landmark is defined as $P_{i}=\left[x_{i},y_{i}\right]$. Based on the detected landmarks, a total of 17 angular features are extracted to quantitatively represent geometric variations in facial components associated with emotional expressions.

#### 3.3.1. Angular Feature Extraction

**Figure 4:**
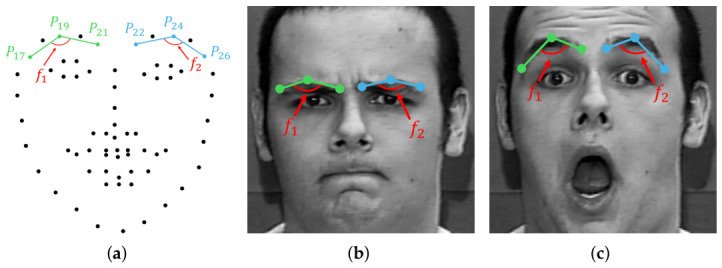
Eyebrow angle features extracted from facial landmarks. (**a**) Landmark configuration used for eyebrow angle extraction. (**b**) Example of eyebrow angles under anger. (**c**) Example of eyebrow angles under surprise.

As shown in Figure 4, eyebrow movement provides important cues for emotional expressions because the shape and curvature of the eyebrows change significantly depending on emotional states. To quantitatively represent the degree of eyebrow elevation or lowering, two angles are computed using the landmark triplets 17–19–21 and 22–24–26, resulting in the features $f_{1}$ and $f_{2}$, respectively.

(1) $$\begin{array}{cc} f_{1} & =\arccos\left(\frac{\langle P_{17}-P_{19},P_{21}-P_{19}\rangle }{∥P_{17}-P_{19}{∥}_{2}{∥P_{21}-P_{19}∥}_{2}}\right), \end{array}$$

(2) $$\begin{array}{cc} f_{2} & =\arccos\left(\frac{\langle P_{22}-P_{24},P_{26}-P_{24}\rangle }{∥P_{22}-P_{24}{∥}_{2}{∥P_{26}-P_{24}∥}_{2}}\right), \end{array}$$

where, 〈,〉 denotes the dot product between two vectors, and ${∥\cdot ∥}_{2}$ represents the Euclidean norm of a vector.

**Figure 5:**
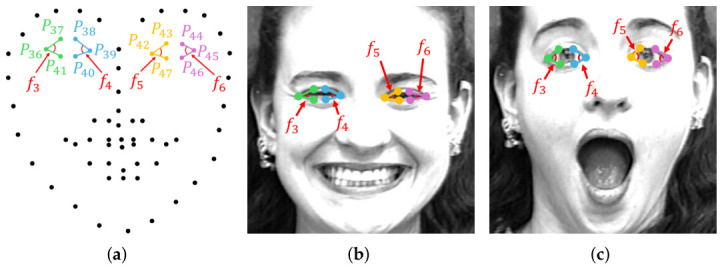
Extraction of eye angular features from facial landmarks. (**a**) Facial landmarks used for eye angle computation. (**b**) Example of eye angle variation under happiness. (**c**) Example of eye angle variation under surprise.

For the eye region, four angular features are computed to represent the curvature and openness of both eyes, which vary depending on emotional expressions. As illustrated in Figure 5, the angles are computed using the landmark triplets 37–36–41, 38–39–40, 43–42–47, and 44–45–46, resulting in the features $f_{3}$, $f_{4}$, $f_{5}$, and $f_{6}$, respectively.

(3) $$\begin{array}{cc} f_{3} & =\arccos\left(\frac{\langle P_{37}-P_{36},P_{41}-P_{36}\rangle }{∥P_{37}-P_{36}{∥}_{2}{∥P_{41}-P_{36}∥}_{2}}\right), \end{array}$$

(4) $$\begin{array}{cc} f_{4} & =\arccos\left(\frac{\langle P_{38}-P_{39},P_{40}-P_{39}\rangle }{∥P_{38}-P_{39}{∥}_{2}{∥P_{40}-P_{39}∥}_{2}}\right), \end{array}$$

(5) $$\begin{array}{cc} f_{5} & =\arccos\left(\frac{\langle P_{43}-P_{42},P_{47}-P_{42}\rangle }{∥P_{43}-P_{42}{∥}_{2}{∥P_{47}-P_{42}∥}_{2}}\right), \end{array}$$

(6) $$\begin{array}{cc} f_{6} & =\arccos\left(\frac{\langle P_{44}-P_{45},P_{46}-P_{45}\rangle }{∥P_{44}-P_{45}{|}_{2}{∥P_{46}-P_{45}∥}_{2}}\right). \end{array}$$

These angular features effectively capture variations in eye shape and openness associated with different emotional states.

**Figure 6:**
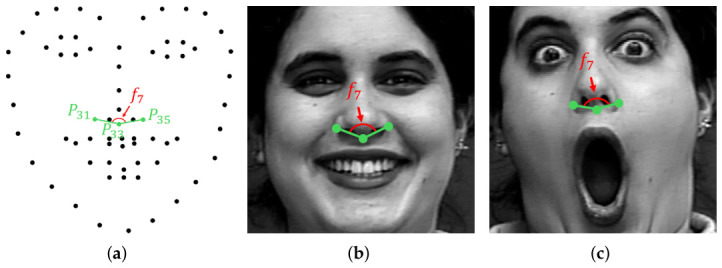
Extraction of nose angular feature from facial landmarks. (**a**) Facial landmarks used for nose angle computation. (**b**) Example of nose angle variation under happiness. (**c**) Example of nose angle variation under surprise.

For the nose region, the angle formed by landmarks 31–33–35 is calculated to represent the degree of nose lifting or tension in the nasal region, as illustrated in Figure 6. This angle is defined as feature $f_{7}$.

(7) $$f_{7}=\arccos\left(\frac{\langle P_{31}-P_{33},P_{35}-P_{33}\rangle }{∥P_{31}-P_{33}{∥}_{2}{∥P_{35}-P_{33}∥}_{2}}\right).$$

This angular feature captures structural variations in the nasal region that may vary across different emotional expressions.

**Figure 7:**
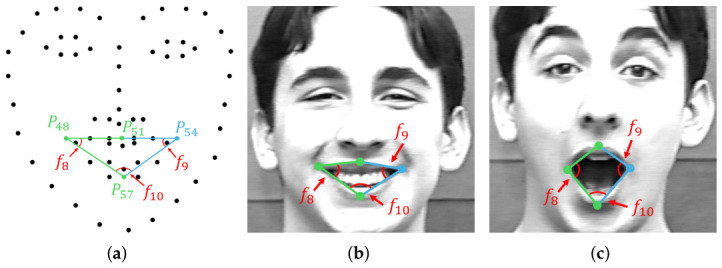
Extraction of outer lip angular features from facial landmarks. (**a**) Facial landmarks used for outer lip angle computation. (**b**) Example of outer lip angle variation under happiness. (**c**) Example of outer lip angle variation under surprise.

The mouth region contains rich structural information related to emotional expressions because mouth shape and lip curvature vary significantly across different emotional states. The outer lip contour is represented using three angular features. Specifically, as illustrated in Figure 7, the landmark triplets 51–48–57, 51–54–57, and 48–57–54 are used to represent the left mouth corner, right mouth corner, and lower lip curvature, respectively. These angles correspond to the features $f_{8}$, $f_{9}$, and $f_{10}$.

(8) $$\begin{array}{cc} f_{8} & =\arccos\left(\frac{\langle P_{51}-P_{48},P_{57}-P_{48}\rangle }{∥P_{51}-P_{48}{∥}_{2}{∥P_{57}-P_{48}∥}_{2}}\right), \end{array}$$

(9) $$\begin{array}{cc} f_{9} & =\arccos\left(\frac{\langle P_{51}-P_{54},P_{57}-P_{54}\rangle }{∥P_{51}-P_{54}{∥}_{2}{∥P_{57}-P_{54}∥}_{2}}\right), \end{array}$$

(10) $$\begin{array}{cc} f_{10} & =\arccos\left(\frac{\langle P_{48}-P_{57},P_{54}-P_{57}\rangle }{∥P_{48}-P_{57}{∥}_{2}{∥P_{54}-P_{57}∥}_{2}}\right). \end{array}$$

These angular features effectively capture variations in mouth shape and lip curvature associated with different emotional expressions.

**Figure 8:**
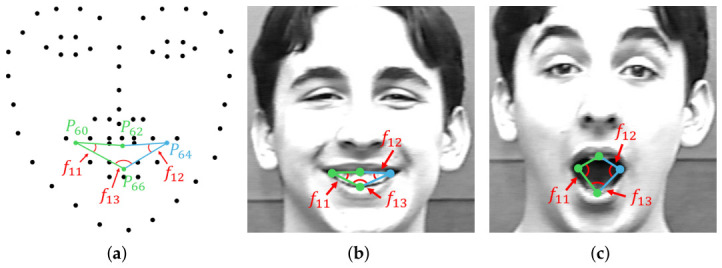
Extraction of inner lip angular features from facial landmarks. (**a**) Facial landmarks used for inner lip angle computation. (**b**) Example of inner lip angle variation under happiness. (**c**) Example of inner lip angle variation under surprise.

The inner lip region is further represented using three angular features computed from the landmark triplets 62–60–66, 62–64–66, and 60–66–64, as illustrated in Figure 8. These angular features describe variations in the curvature and openness of the inner lip structure associated with emotional expressions, and correspond to the features $f_{11}$, $f_{12}$, and $f_{13}$, respectively.

(11) $$\begin{array}{cc} f_{11} & =\arccos\left(\frac{\langle P_{62}-P_{60},P_{66}-P_{60}\rangle }{∥P_{62}-P_{60}{∥}_{2}{∥P_{66}-P_{60}∥}_{2}}\right), \end{array}$$

(12) $$\begin{array}{cc} f_{12} & =\arccos\left(\frac{\langle P_{62}-P_{64},P_{66}-P_{64}\rangle }{∥P_{62}-P_{64}{∥}_{2}{∥P_{66}-P_{64}∥}_{2}}\right), \end{array}$$

(13) $$\begin{array}{cc} f_{13} & =\arccos\left(\frac{\langle P_{60}-P_{66},P_{64}-P_{66}\rangle }{∥P_{60}-P_{66}{∥}_{2}{∥P_{64}-P_{66}∥}_{2}}\right). \end{array}$$

**Figure 9:**
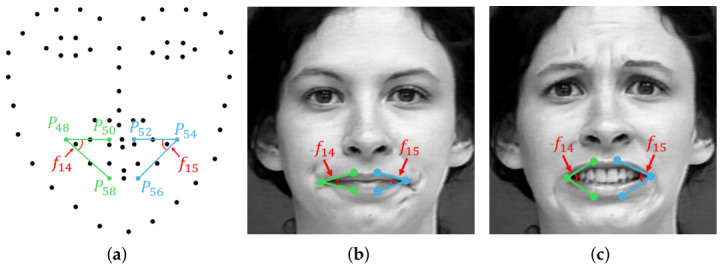
Extraction of mouth corner angular features from facial landmarks. (**a**) Facial landmarks used for mouth corner angle computation. (**b**) Example of mouth corner angle variation under contempt. (**c**) Example of mouth corner angle variation under fear.

To capture subtle geometric variations around the mouth corners, two additional angular features are computed from the landmark triplets 50–48–58 and 52–54–56, as illustrated in Figure 9. These features represent local mouth corner deformations and asymmetrical facial movements associated with emotional expressions, and correspond to the features $f_{14}$ and $f_{15}$, respectively.

(14) $$\begin{array}{cc} f_{14} & =\arccos\left(\frac{\langle P_{50}-P_{48},P_{58}-P_{48}\rangle }{∥P_{50}-P_{48}{∥}_{2}{∥P_{58}-P_{48}∥}_{2}}\right), \end{array}$$

(15) $$\begin{array}{cc} f_{15} & =\arccos\left(\frac{\langle P_{52}-P_{54},P_{56}-P_{54}\rangle }{∥P_{52}-P_{54}{∥}_{2}{∥P_{56}-P_{54}∥}_{2}}\right). \end{array}$$

These angular features effectively capture localized mouth corner movements that frequently occur in expressive facial behaviors.

**Figure 10:**
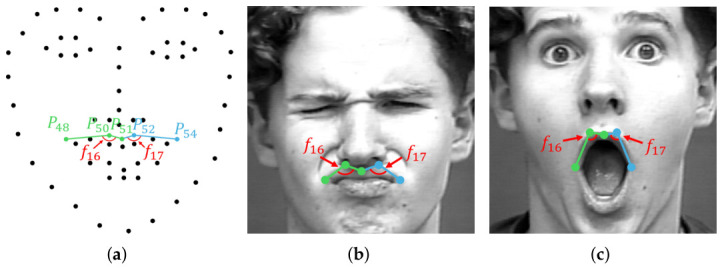
Extraction of cupid’s bow angular features from facial landmarks. (**a**) Facial landmarks used for cupid’s bow angle computation. (**b**) Example of cupid’s bow angle variation under disgust. (**c**) Example of cupid’s bow angle variation under surprise.

Finally, two angular features are computed to capture asymmetrical movements and localized geometric deformations around the cupid’s bow region of the lips. As illustrated in Figure 10, these angles are calculated using the landmark triplets 48–50–51 and 51–52–54, producing the features $f_{16}$ and $f_{17}$, respectively.

(16) $$\begin{array}{cc} f_{16} & =\arccos\left(\frac{\langle P_{48}-P_{50},P_{51}-P_{50}\rangle }{∥P_{48}-P_{50}{∥}_{2}{∥P_{51}-P_{50}∥}_{2}}\right), \end{array}$$

(17) $$\begin{array}{cc} f_{17} & =\arccos\left(\frac{\langle P_{51}-P_{52},P_{54}-P_{52}\rangle }{∥P_{51}-P_{52}{∥}_{2}{∥P_{54}-P_{52}∥}_{2}}\right). \end{array}$$

The proposed angular features were designed to capture localized geometric variations that are closely associated with facial expressions while remaining robust to individual differences in facial morphology. Unlike distance- or ratio-based geometric descriptors, angle-based features represent the relative structural relationships among facial landmarks and are inherently less sensitive to facial scale, image resolution, and inter-subject facial morphology. Since facial expressions are primarily characterized by changes in eyebrow inclination, eye opening, mouth corner movement, and lip deformation, angular features provide an effective representation of these localized geometric variations while reducing the influence of individual facial shape differences.

Consequently, the selected angular features effectively describe geometric deformations occurring in the major facial components involved in facial expressions, including the eyebrows, eyes, nose, and mouth, while providing complementary geometric information for CNN-based deep representations.

#### 3.3.2. HOG Feature Extraction

**Figure 11:**
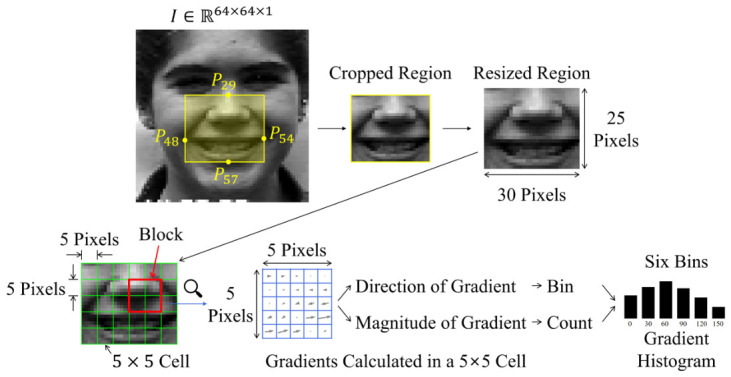
Extraction process of HOG features from the nose and mouth regions. The arrows indicate the sequential processing pipeline, where the facial region is cropped based on facial landmarks, resized to a fixed resolution, and subsequently divided into cells and blocks for gradient histogram computation.

HOG is used to capture local edge and contour information from facial regions. The overall procedure for extracting HOG features from the selected facial regions is illustrated in Figure 11. These gradient-based features are particularly effective for representing subtle variations in facial expressions. First, the gradient magnitude and orientation are computed for each pixel in the image. In this study, the number of orientation bins is set to six, dividing the gradient range of 0–180° into intervals of 30°.

The image is then divided into cells of size 5×5 pixels, and a histogram of gradient orientations is computed for each cell. To improve robustness against illumination variations and local intensity changes, neighboring cells are grouped into blocks consisting of 2×2 cells, and block-wise normalization is performed.

Since the nose and mouth regions exhibit significant appearance changes during facial expressions, these regions are selected for HOG feature extraction. Specifically, the horizontal boundaries are defined by landmarks 48 and 54, while the vertical boundaries are defined by landmarks 29 and 57. The cropped region is then resized to 25×30 pixels to ensure consistency across different samples.

With this configuration, a total of 30 cells (5×6) and 20 blocks (4×5) are generated. Each block consists of four cells, and the feature dimension for each block is calculated as 6 orientations multiplied by 4 cells, resulting in 24 features per block. Consequently, the final HOG descriptor is represented as a 480-dimensional feature vector.

To further justify the selection of the nose and mouth regions for HOG feature extraction, gradient-weighted class activation mapping (Grad-CAM) analysis was conducted using the baseline LeNet-5 model. The activation maps consistently indicated that the CNN primarily focuses on the central and lower facial regions, particularly around the nose and mouth. Representative Grad-CAM activation maps are shown in Figure S1 of the Supplementary Materials, further supporting the selection of these facial regions for HOG feature extraction.

### 3.4. Feature Fusion and Emotion Classification

Deep features are extracted using a CNN, which is effective for learning hierarchical feature representations from facial images. The CNN model consists of multiple convolutional and pooling layers. The convolutional layers learn local spatial patterns such as edges, textures, and facial structures, while the pooling layers reduce spatial dimensions and improve robustness against small variations in facial appearance. After the convolution and pooling operations, the resulting feature maps are flattened to generate a deep feature vector representing high-level semantic information related to facial expressions.

**Figure 12:**
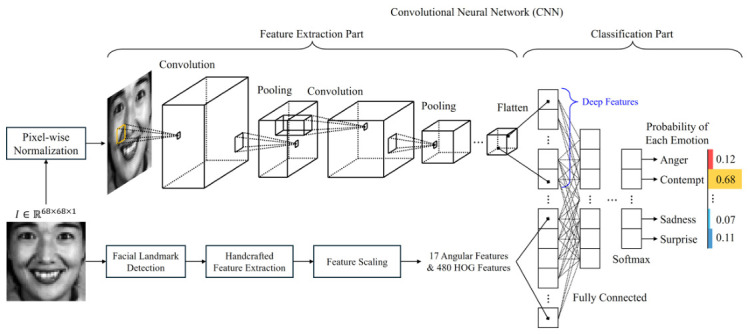
Overall architecture of the proposed feature fusion framework combining CNN-based deep features and handcrafted features for facial emotion recognition.

As illustrated in Figure 12, the proposed method combines the deep feature vector extracted from the CNN model with handcrafted features, including the proposed angular features and HOG features. Prior to feature fusion, the handcrafted features are normalized through feature scaling to reduce differences in feature magnitude and improve stable learning behavior. The handcrafted features are then concatenated with the deep feature vector obtained from the flatten layer of the CNN model. This concatenation produces a unified feature representation that integrates both high-level semantic representations and explicit geometric characteristics of facial expressions.

The fused feature vector is subsequently passed through fully connected layers for emotion classification. Finally, a softmax activation function is applied to generate the probability distribution over the emotion classes, and the class with the highest probability is selected as the final prediction result.

## 4. Experimental Setup

To comprehensively evaluate the proposed facial emotion recognition framework, experiments were conducted using three controlled facial emotion recognition datasets (JAFFE, CK+, and KDEF) together with two large-scale in-the-wild datasets (RAF-DB and AffectNet). The controlled datasets were used to evaluate the effectiveness of the proposed feature-fusion strategy under laboratory conditions, whereas the large-scale datasets were employed to assess its generalization capability under realistic facial emotion recognition scenarios.

### 4.1. Datasets

#### 4.1.1. JAFFE

The Japanese Female Facial Expression (JAFFE) dataset [32] consists of facial images collected from ten Japanese female subjects. Following previous studies, six emotion categories were considered in this study: anger (AN), disgust (DI), fear (FE), happiness (HA), sadness (SA), and surprise (SU), resulting in a total of 183 facial images. The dataset provides well-controlled frontal facial images with relatively limited intra-class variations, making it suitable for evaluating facial emotion recognition methods under laboratory conditions. Following previous studies, the neutral expression category was excluded from the experimental evaluation. The primary objective of this study is to distinguish facial expressions characterized by explicit geometric deformation and local texture variation. Since the neutral class represents a reference facial state with minimal facial deformation rather than a distinct emotional expression, it provides relatively limited discriminative information for the proposed landmark-based angular features and nose/mouth HOG features. Consequently, only the six non-neutral emotion categories were considered throughout this study.

#### 4.1.2. CK+

The Extended Cohn–Kanade (CK+) dataset [33] consists of facial expression image sequences collected from 210 subjects with diverse demographic characteristics. Following the standard experimental protocol, only the peak-expression images were used in this study. The dataset contains seven emotion categories: anger (AN), contempt (CO), disgust (DI), fear (FE), happiness (HA), sadness (SA), and surprise (SU), resulting in a total of 327 labeled facial images. Compared with JAFFE, the CK+ dataset provides greater subject diversity and more expressive facial deformations while maintaining controlled imaging conditions.

#### 4.1.3. KDEF

The Karolinska Directed Emotional Faces (KDEF) dataset [34] contains facial images collected from 70 subjects (35 males and 35 females) captured multiple viewing angles. In this study, only frontal-view images were used because reliable facial landmark localization and region-based HOG feature extraction require near-frontal facial alignment. Six emotion categories were considered: afraid (AF), anger (AN), disgust (DI), happiness (HA), sadness (SA), and surprise (SU), resulting in a total of 840 facial images. Compared with the JAFFE and CK+ datasets, KDEF provides a larger number of subjects while preserving controlled acquisition conditions. For consistency throughout this paper, the emotion category originally annotated as “afraid” in the KDEF dataset is referred to as “fear” (FE), following the terminology adopted for the other datasets.

#### 4.1.4. RAF-DB

The Real-world Affective Faces Database (RAF-DB) [35] is a large-scale in-the-wild facial emotion recognition dataset containing facial images collected from the Internet under diverse real-world conditions. In this study, the official train/test split provided by the dataset was adopted. The dataset includes seven basic emotion categories: anger (AN), disgust (DI), fear (FE), happiness (HA), sadness (SA), surprise (SU), and neutral (NE). Following the experimental settings adopted in previous studies, only the six non-neutral emotion categories were considered for evaluation. Compared with the controlled datasets, RAF-DB contains substantially greater variations in facial pose, illumination, occlusion, expression intensity, and background, providing a more challenging benchmark for evaluating the robustness of facial emotion recognition methods.

#### 4.1.5. AffectNet

The AffectNet dataset [36] is one of the largest publicly available facial emotion recognition datasets, containing facial images collected from the Internet under highly diverse real-world conditions. The images exhibit substantial variations in facial pose, illumination, occlusion, expression intensity, ethnicity, age, and imaging quality, making AffectNet one of the most challenging benchmarks for facial emotion recognition. In this study, the official train/test split provided by the dataset was adopted. The dataset contains eight manually annotated emotion categories. Following the experimental protocol adopted in this study, only the seven non-neutral emotion categories, namely anger (AN), contempt (CO), disgust (DI), fear (FE), happiness (HA), sadness (SA), and surprise (SU), were used for performance evaluation. Compared with the other datasets considered in this study, AffectNet provides the largest scale and the highest degree of real-world variability, enabling a comprehensive evaluation of the robustness and generalization capability of the proposed facial emotion recognition framework.

Overall, the five datasets considered in this study provide complementary evaluation scenarios, ranging from controlled laboratory environments (JAFFE, CK+, and KDEF) to large-scale in-the-wild facial emotion recognition benchmarks (RAF-DB and AffectNet). The dataset configurations and evaluation protocols adopted in this study follow the corresponding benchmark settings and are consistent with those commonly used in previous facial emotion recognition studies. Consequently, these datasets enable a comprehensive assessment of the proposed method in terms of recognition accuracy, subject-independent generalization, and robustness under realistic operating conditions.

### 4.2. Data Augmentation

To alleviate the limited amount of training data and reduce class imbalance, CAE-based data augmentation was employed. The CAE was applied exclusively to the training data, whereas the validation and test sets remained completely unchanged throughout all experiments to prevent information leakage. Consequently, no augmented samples were included in the validation or test sets.

The trained CAE was used to generate additional facial images only for the training set in each experimental split or cross-validation fold. The number of generated images for each emotion category was determined according to the class distribution of the corresponding dataset in order to alleviate class imbalance while preserving the original test distribution.

The detailed architecture and training configuration of the adopted CAE are provided in the Supplementary Materials (Section S2) for reproducibility.

### 4.3. Experimental Protocol

To comprehensively evaluate the effectiveness, robustness, and generalization capability of the proposed method, three complementary evaluation protocols were adopted. Five-fold cross-validation was used to evaluate the robustness of the proposed method under different data partitions, LOSO cross-validation was employed to assess subject-independent generalization, and the official train/test splits of the RAF-DB and AffectNet datasets were adopted to evaluate the proposed method under large-scale in-the-wild conditions.

#### 4.3.1. Five-Fold Cross-Validation

Five-fold cross-validation was conducted on the JAFFE, CK+, and KDEF datasets to obtain a statistically reliable evaluation of the proposed method. Stratified sampling was adopted to preserve the class distribution across all folds. For each fold, the training subset was further divided into training and validation sets with a ratio of 8:2. The validation set was used exclusively for model selection and early stopping, whereas the corresponding test fold was used only for final performance evaluation.

To prevent information leakage, all preprocessing procedures were independently performed within each fold. Specifically, CAE-based data augmentation was applied only to the training subset. Likewise, principal component analysis (PCA), feature normalization, and feature scaling were fitted exclusively on the training data and subsequently applied to the validation and test data using the corresponding fitted models. The reported performance was obtained by averaging the results over all five folds together with the corresponding standard deviations.

#### 4.3.2. Leave-One-Subject-Out Cross-Validation

LOSO cross-validation was additionally performed on the JAFFE and KDEF datasets to evaluate the subject-independent generalization capability of the proposed method. In each iteration, all facial images belonging to one subject were reserved for testing, whereas the remaining subjects were used for model training and validation.

LOSO evaluation was not conducted on the CK+ dataset because the subset of peak-expression images used in this study does not provide a sufficiently balanced distribution of emotion categories for every subject. Specifically, some subjects contain only a limited subset of emotion classes, making subject-independent evaluation unreliable and potentially introducing substantial class imbalance in the training and test partitions.

#### 4.3.3. Official Train/Test Split Evaluation

For the RAF-DB and AffectNet datasets, the official train/test splits provided by the dataset authors were adopted to facilitate direct comparison with previous studies. No additional data partitioning was performed. Similar to the cross-validation protocols, all preprocessing procedures, including CAE-based data augmentation, PCA, feature normalization, and feature scaling, were applied exclusively to the training data. The trained models were subsequently evaluated using the corresponding official test sets without any additional fitting or parameter adjustment.

The three evaluation protocols provide complementary assessments of the proposed method in terms of robustness, subject-independent generalization, and performance under realistic large-scale facial emotion recognition scenarios.

### 4.4. Benchmark Methods

To comprehensively evaluate the effectiveness of the proposed feature-fusion strategy, three CNN backbone networks with different model complexities were considered: LeNet-5, ResNet-18, and MobileNetV2. LeNet-5 was selected because its relatively lightweight architecture is suitable for small-scale facial emotion recognition datasets and is less prone to overfitting. ResNet-18 was adopted as a representative residual learning architecture, whereas MobileNetV2 was included as a lightweight CNN designed for computationally efficient visual recognition.

For all backbone networks, deep features were extracted immediately before the final classification layer and subsequently fused with the proposed handcrafted features through feature-level concatenation. The handcrafted features consisted of the proposed landmark-based angular features and HOG features. Since the HOG descriptor has a relatively high dimensionality, PCA was applied to the HOG features while retaining 90% of the cumulative explained variance to reduce feature redundancy and computational complexity.

To investigate the individual contribution of each handcrafted feature type, four feature-fusion settings were considered. The corresponding feature combinations are summarized in Table 1.

As shown in Table 1, Bench. 1 represents the CNN-only baseline, whereas Bench. 2 and Bench. 3 evaluate the individual contribution of the proposed angular and HOG features, respectively. The proposed method combines all feature types to investigate the effectiveness of the complete feature-fusion strategy.

### 4.5. Implementation Details

All experiments were implemented using Python 3.12.13 and PyTorch 2.11.0 (CUDA 12.8). Detailed software versions, hardware specifications, training hyperparameters, and reproducibility settings are provided in the Supplementary Materials (Section S3). Facial regions were detected using the Dlib frontal face detector, and facial landmarks were extracted using the Dlib 68-point facial landmark predictor. HOG features were extracted from the nose and mouth regions, followed by PCA with 90% cumulative explained variance retention.

For all CNN backbone networks, the Adam optimizer and categorical cross-entropy loss function were adopted. During five-fold cross-validation, the training subset was further divided into training and validation sets for early stopping. To ensure experimental reproducibility, all random seeds were fixed, and deterministic CuDNN settings were adopted throughout all experiments.

### 4.6. Evaluation Metrics

For performance evaluation, the macro-average true positive rate (macro-TPR), macro-average positive predictive value (macro-PPV), macro-average F1-score (macro-F1), and overall accuracy were used. These evaluation metrics were selected to provide a balanced assessment of classification performance, particularly for datasets with class imbalance.

The true positive rate (TPR), also referred to as recall or sensitivity, represents the proportion of correctly classified positive samples among all actual positive samples. TPR is defined as follows:

(18) $$TPR=\frac{TP}{TP+FN},$$

where TP denotes the number of true positive samples and FN denotes the number of false negative samples.

The positive predictive value (PPV), also referred to as precision, indicates the proportion of correctly predicted positive samples among all samples predicted as positive. PPV is computed as follows:

(19) $$PPV=\frac{TP}{TP+FP},$$

where FP represents the number of false positive samples.

The F1-score is defined as the harmonic mean of TPR and PPV, providing a balanced evaluation between recall and precision. The F1-score is calculated as follows:

(20) $$F1=\frac{2\times PPV\times TPR}{PPV+TPR}.$$

For multi-class emotion recognition, macro-average metrics were adopted because the datasets used in this study exhibit class imbalance. Macro-average evaluation computes the arithmetic mean of the class-wise metric values, assigning equal importance to all emotion classes regardless of the number of samples. The macro-average metric is defined as follows:

(21) $$Macro-Metric=\frac{1}{C}\sum_{i=1}^{C}Metric_{i},$$

where *C* denotes the total number of emotion classes and $Metric_{i}$ represents the evaluation value of the *i*-th class.

In addition, the overall classification accuracy was used to evaluate the general recognition performance of the proposed method. Accuracy is defined as follows:

(22) $$Accuracy=\frac{TP+TN}{TP+TN+FP+FN},$$

where TN denotes the number of true negative samples. By combining macro-average metrics and overall accuracy, the proposed method can be evaluated from both class-balanced and overall classification perspectives.

## 5. Results Under Five-Fold Cross-Validation

### 5.1. Results on the JAFFE Dataset

#### 5.1.1. Ablation Study

Table 2 summarizes the quantitative results of the ablation study under five-fold cross-validation on the JAFFE dataset using three different CNN backbones. For each backbone, four feature settings were evaluated to investigate the contribution of the proposed handcrafted features.

For the LeNet-5 backbone, the proposed method achieved the highest overall performance, obtaining a macro-F1 score of 0.791±0.024 and an accuracy of 0.758±0.041. Compared with Bench. 1, which uses only CNN-based deep features, both Bench. 2 and Bench. 3 consistently improved the recognition performance, indicating that landmark-based angular features and HOG features each provide complementary information for facial emotion recognition. Combining both handcrafted feature types further improved the overall performance, demonstrating their complementary contribution.

A similar tendency was observed for the ResNet-18 and MobileNetV2 backbones. In particular, the proposed method consistently outperformed the corresponding benchmark settings for both networks. For ResNet-18, the proposed method improved the accuracy from 0.703±0.085 (Bench. 1) to 0.762±0.025, while the macro-F1 score increased from 0.717±0.090 to 0.770±0.030. Likewise, for MobileNetV2, the proposed method achieved the highest performance among the four feature settings, confirming that the effectiveness of the proposed feature-fusion strategy is maintained across different CNN architectures.

Overall, these results demonstrate that the proposed handcrafted features provide complementary information to CNN-based deep representations regardless of the backbone network. The consistent improvements observed across lightweight (LeNet-5), residual (ResNet-18), and mobile-oriented (MobileNetV2) architectures indicate that the proposed feature-fusion strategy is not specific to a particular CNN model and can be effectively integrated with diverse backbone networks.

To provide a more detailed class-wise analysis, the complete per-class performance results for all backbone networks are provided in the Supplementary Materials (Section S4.1.1).

Table 3 summarizes the training and inference time of the benchmark methods and the proposed method under five-fold cross-validation on the JAFFE dataset. For brevity, conventional handcrafted feature-based methods, standard CNN models, transfer learning-based models, and hybrid feature-fusion methods are denoted as CH, CNN, TL, and HF, respectively, throughout the remainder of this paper.

For all three backbone networks, incorporating additional handcrafted features slightly increased both the training and inference time compared with the CNN-only baseline (Bench. 1). This increase is mainly attributed to the additional computation required for handcrafted feature extraction and feature concatenation prior to classification. Nevertheless, the increase in computational cost remained relatively small for all backbone architectures.

Among the evaluated backbones, LeNet-5 required the shortest training time owing to its lightweight architecture, whereas ResNet-18 required the longest training time because of its substantially larger network capacity. MobileNetV2 required substantially less training time than ResNet-18, reflecting its lightweight architecture, although ResNet-18 generally achieved higher recognition performance.

Although the proposed method introduces additional computational overhead compared with the benchmark settings, the increase in computational cost is relatively small compared with the consistent improvement in recognition performance observed in Table 2. Moreover, the inference time remained below 0.1 ms/image for all evaluated backbone networks, suggesting that the proposed feature-fusion strategy is computationally feasible for practical facial emotion recognition applications. Overall, these results indicate that the proposed feature-fusion strategy provides an effective trade-off between recognition accuracy and computational efficiency.

#### 5.1.2. Statistical Analysis

Paired *t*-tests were performed to evaluate the statistical significance of the observed performance differences between the benchmark methods and the proposed method. The complete statistical analysis results for each backbone network and evaluation protocol are provided in the Supplementary Materials (Section S4.1.2).

#### 5.1.3. Comparison with Existing Methods

Table 4 presents a performance comparison between the proposed method and representative facial emotion recognition approaches from four benchmark categories, namely conventional handcrafted feature-based methods (CH), standard CNN models (CNN), transfer learning-based models (TL), and hybrid feature-fusion methods (HF), under five-fold cross-validation on the JAFFE dataset.

Among the CH methods, the proposed method substantially outperformed the existing approaches across all evaluation metrics. Compared with the representative baseline CNN models, the proposed method consistently demonstrated competitive recognition performance, indicating that the integration of handcrafted angular and HOG features effectively complements CNN-based deep representations. In particular, the proposed method achieved the highest overall accuracy when using the ResNet-18 backbone (0.762±0.025), whereas the highest macro-F1 score was obtained using the LeNet-5 backbone (0.791±0.024).

The proposed method also showed competitive performance compared with transfer learning-based methods and recent hybrid feature-fusion approaches. Although VGG-19 achieved a comparable macro-F1 score, the proposed method consistently provided higher macro-average recall and overall accuracy while using substantially lighter backbone architectures. The relatively lower performance of ResNet-152 is likely related to its substantially larger model capacity, which makes optimization more challenging on the relatively small JAFFE dataset despite employing transfer learning. Overall, these comparative results demonstrate that the proposed feature-fusion strategy is not only effective for lightweight CNN backbones but also remains competitive with representative conventional, transfer learning-based, and hybrid facial emotion recognition approaches, further validating its general applicability across diverse facial emotion recognition frameworks.

### 5.2. Results on the CK+ Dataset

#### 5.2.1. Ablation Study

Table 5 presents the performance comparison of the benchmark methods and the proposed method under five-fold cross-validation on the CK+ dataset using three different CNN backbones.

For both the LeNet-5 and ResNet-18 backbones, the proposed method consistently achieved the best overall performance among the four feature settings. Compared with the CNN-only baseline (Bench. 1), the proposed method improved the accuracy from 0.655±0.015 to 0.823±0.028 for LeNet-5 and from 0.618±0.068 to 0.813±0.046 for ResNet-18. Similar improvements were also observed for the macro-average TPR, PPV, and F1-score, demonstrating that the proposed combination of angular and HOG features effectively complements the CNN-based deep representations. These results indicate that progressively incorporating handcrafted features consistently improved the recognition performance, with the proposed method achieving the best overall performance for both the LeNet-5 and ResNet-18 backbones.

For the MobileNetV2 backbone, the proposed method also achieved the highest overall performance, although the improvement over the benchmark settings was relatively modest. This result suggests that the effectiveness of the proposed handcrafted features is influenced by the representation capability of the underlying CNN backbone, while still providing consistent performance gains across different architectures.

Overall, the results obtained on the CK+ dataset are consistent with those observed for the JAFFE dataset, further confirming that the proposed feature-fusion strategy generalizes well across different CNN backbones and controlled facial emotion recognition datasets.

To provide a more detailed class-wise analysis, the complete per-class performance results for all backbone networks are provided in the Supplementary Materials (Section S4.2.1).

Table 6 summarizes the computational efficiency of the benchmark methods and the proposed method in terms of training and inference time under five-fold cross-validation on the CK+ dataset. Similar to the observations on the JAFFE dataset, incorporating additional handcrafted features gradually increased both the training and inference time for all evaluated backbone networks because of the additional computation required for handcrafted feature extraction and feature fusion. Nevertheless, the additional computational overhead remained relatively small compared with the corresponding improvement in recognition performance.

Among the evaluated backbone networks, LeNet-5 required the shortest training and inference time owing to its lightweight architecture, whereas ResNet-18 exhibited the highest computational cost because of its substantially larger network capacity. MobileNetV2 required considerably less training time than ResNet-18 while maintaining competitive recognition performance, demonstrating the efficiency advantage of lightweight CNN architectures.

Overall, the results on the CK+ dataset are consistent with those obtained on the JAFFE dataset, indicating that the proposed feature-fusion strategy provides an effective balance between recognition accuracy and computational efficiency across different CNN backbones.

#### 5.2.2. Statistical Analysis

Paired *t*-tests were performed to evaluate the statistical significance of the observed performance differences between the benchmark methods and the proposed method. The complete statistical analysis results for each backbone network and evaluation protocol are provided in the Supplementary Materials (Section S4.2.2).

#### 5.2.3. Comparison with Existing Methods

Table 7 presents the performance comparison between the proposed method and representative facial emotion recognition approaches under five-fold cross-validation on the CK+ dataset, including conventional handcrafted feature-based methods (CH), standard CNN models (CNN), transfer learning-based models (TL), and hybrid feature-fusion methods (HF).

Across all benchmark categories, the proposed method consistently achieved competitive or superior recognition performance. Compared with the CH methods and representative CNN models, the proposed method substantially improved all evaluation metrics, demonstrating that the proposed combination of angular and HOG features effectively complements CNN-based deep representations. In particular, the proposed method achieved the highest overall accuracy of 0.823±0.028 when using the LeNet-5 backbone, whereas the highest macro-F1 score of 0.805±0.025 was obtained using the ResNet-18 backbone.

The proposed method also outperformed the representative transfer learning-based and hybrid feature-fusion approaches. Although VGG-19 achieved competitive recognition performance, the proposed method consistently provided higher overall accuracy and macro-average performance while employing considerably lighter backbone architectures. Similar to the observations on the JAFFE dataset, the relatively lower performance of ResNet-152 is likely related to its substantially larger model capacity, which makes optimization more challenging on the relatively small CK+ dataset despite employing transfer learning.

Overall, these comparative results further demonstrate that the proposed feature-fusion strategy generalizes well across different benchmark categories and CNN backbones, consistently providing competitive facial emotion recognition performance on the CK+ dataset.

### 5.3. Results on the KDEF Dataset

#### 5.3.1. Ablation Study

Table 8 presents the quantitative performance comparison of the benchmark methods and the proposed method under five-fold cross-validation on the KDEF dataset using three different CNN backbones.

For all evaluated backbone networks, the proposed method consistently achieved the highest overall recognition performance among the four feature settings. Compared with the CNN-only baseline (Bench. 1), substantial improvements were observed for the LeNet-5 and ResNet-18 backbones. In particular, the proposed method improved the accuracy from 0.702±0.025 to 0.843±0.023 for LeNet-5 and from 0.710±0.036 to 0.821±0.028 for ResNet-18. Similar improvements were also observed for the macro-average TPR, PPV, and F1-score, demonstrating the effectiveness of integrating handcrafted angular and HOG features with CNN-based deep representations.

For the MobileNetV2 backbone, the proposed method also achieved the best overall performance, although the improvement over the benchmark settings was relatively modest. Nevertheless, the proposed method consistently outperformed the corresponding benchmark configurations across all evaluation metrics, indicating that the proposed feature-fusion strategy remains effective even for lightweight CNN architectures.

Overall, the results obtained on the KDEF dataset are consistent with those observed on the JAFFE and CK+ datasets, further confirming that the proposed feature-fusion strategy provides stable and consistent performance improvements across different controlled facial emotion recognition datasets and CNN backbones.

To provide a more detailed class-wise analysis, the complete per-class performance results for all backbone networks are provided in the Supplementary Materials (Section S4.3.1).

Table 9 summarizes the computational efficiency of the benchmark methods and the proposed method in terms of training and inference time under five-fold cross-validation on the KDEF dataset. Consistent with the observations on the JAFFE and CK+ datasets, incorporating additional handcrafted features gradually increased both the training and inference time for all evaluated backbone networks. This increase is primarily attributed to the additional computation required for handcrafted feature extraction and feature fusion. Nevertheless, the additional computational overhead remained relatively small compared with the corresponding improvement in recognition performance.

Among the evaluated backbone networks, LeNet-5 required the shortest training and inference time owing to its lightweight architecture, whereas ResNet-18 exhibited the highest computational cost because of its substantially larger network capacity. MobileNetV2 again provided a favorable balance between computational efficiency and recognition performance, requiring considerably less training time than ResNet-18 while maintaining competitive recognition accuracy.

Overall, the computational efficiency results obtained on the KDEF dataset are consistent with those observed on the JAFFE and CK+ datasets, demonstrating that the proposed feature-fusion strategy exhibits stable computational characteristics while maintaining an effective balance between recognition performance and computational cost across different CNN backbones.

#### 5.3.2. Statistical Analysis

Paired *t*-tests were performed to evaluate the statistical significance of the observed performance differences between the benchmark methods and the proposed method. The complete statistical analysis results for each backbone network and evaluation protocol are provided in the Supplementary Materials (Section S4.3.2).

#### 5.3.3. Comparison with Existing Methods

Table 10 presents the performance comparison of the proposed method with representative facial emotion recognition approaches under five-fold cross-validation on the KDEF dataset, including conventional handcrafted feature-based methods (CH), standard CNN models (CNN), transfer learning-based models (TL), and hybrid feature-fusion methods (HF).

Across all benchmark categories, the proposed method consistently achieved superior recognition performance. Compared with the CH methods and representative CNN models, the proposed method substantially improved all evaluation metrics, demonstrating that the proposed combination of angular and HOG features effectively complements CNN-based deep representations. In particular, the proposed method achieved the highest overall accuracy of 0.843±0.023 when using the LeNet-5 backbone, while the ResNet-18 backbone achieved the highest macro-average TPR and PPV.

The proposed method also outperformed the representative transfer learning-based and hybrid feature-fusion approaches. In particular, the proposed LeNet-5 backbone substantially outperformed VGG-19, demonstrating that the proposed feature-fusion strategy can effectively compensate for the limited representation capability of lightweight CNN architectures. Similar to the observations on the JAFFE and CK+ datasets, the relatively lower performance of ResNet-152 is likely related to its substantially larger model capacity, which makes optimization more challenging on relatively small facial expression datasets despite employing transfer learning.

Overall, these comparative results further demonstrate that the proposed feature-fusion strategy consistently provides competitive or superior performance across different benchmark categories and CNN backbones, confirming its robustness and general applicability for facial emotion recognition.

## 6. Results Under Leave-One-Subject-Out Cross-Validation

LOSO cross-validation was additionally performed on the JAFFE and KDEF datasets to evaluate the subject-independent generalization capability of the proposed method. These datasets provide suitable subject-wise distributions for LOSO evaluation. Although the CK+ dataset contains subject identifiers, the subset of peak-expression images used in this study does not provide a sufficiently balanced distribution of emotion classes for every subject. Consequently, LOSO evaluation was not conducted on the CK+ dataset.

### 6.1. Results on the JAFFE Dataset

#### 6.1.1. Ablation Study

Table 11 presents the quantitative performance comparison of the benchmark methods and the proposed method under LOSO cross-validation on the JAFFE dataset. Compared with the five-fold cross-validation results, the overall recognition performance decreased for all methods because LOSO evaluation requires the model to recognize facial expressions from previously unseen subjects, making the classification task considerably more challenging.

Despite this more rigorous evaluation protocol, the proposed method consistently achieved the best overall performance across all evaluated backbone networks. For the LeNet-5 backbone, the proposed method improved the accuracy from 0.652±0.142 for the CNN-only baseline (Bench. 1) to 0.810±0.170. Similar improvements were also observed for the ResNet-18 and MobileNetV2 backbones, where the proposed method achieved the highest macro-average TPR, PPV, F1-score, and accuracy among the evaluated feature settings.

These results demonstrate that the proposed feature-fusion strategy effectively improves subject-independent facial emotion recognition by providing complementary geometric and texture information that generalizes well to previously unseen subjects.

Table 12 summarizes the computational efficiency of the benchmark methods and the proposed method in terms of training and inference time under LOSO cross-validation on the JAFFE dataset. Consistent with the observations obtained under five-fold cross-validation, incorporating additional handcrafted features gradually increased both the training and inference time for all evaluated backbone networks because of the additional computation required for handcrafted feature extraction and feature fusion. Nevertheless, the resulting computational overhead remained relatively small compared with the corresponding improvement in subject-independent recognition performance.

Among the evaluated backbone networks, LeNet-5 again required the shortest training and inference time owing to its lightweight architecture, whereas ResNet-18 exhibited the highest computational cost because of its substantially larger network capacity. MobileNetV2 required considerably less training time than ResNet-18 while maintaining competitive subject-independent recognition performance, demonstrating the efficiency advantage of lightweight CNN architectures.

Overall, the computational efficiency observed under LOSO cross-validation is consistent with that obtained under five-fold cross-validation, indicating that the proposed feature-fusion strategy maintains a favorable balance between subject-independent recognition performance and computational efficiency.

#### 6.1.2. Statistical Analysis

Paired *t*-tests were performed to evaluate the statistical significance of the observed performance differences between the benchmark methods and the proposed method. The complete statistical analysis results for each backbone network and evaluation protocol are provided in the Supplementary Materials (Section S5.1).

### 6.2. Results on the KDEF Dataset

#### 6.2.1. Ablation Study

Table 13 presents the quantitative performance comparison of the benchmark methods and the proposed method under LOSO cross-validation on the KDEF dataset. Compared with five-fold cross-validation, LOSO evaluation provides a more challenging subject-independent validation protocol by requiring the model to recognize facial expressions from previously unseen subjects.

Despite the increased difficulty of the evaluation protocol, the proposed method consistently achieved the highest overall recognition performance across all evaluated backbone networks. For the LeNet-5 backbone, the proposed method improved the accuracy from 0.666±0.162 for the CNN-only baseline (Bench. 1) to 0.802±0.125. Even larger improvements were observed for the ResNet-18 backbone, where the accuracy increased from 0.601±0.108 to 0.770±0.125, accompanied by consistent improvements in the macro-average TPR, PPV, and F1-score.

For the MobileNetV2 backbone, the proposed method also achieved the best overall performance, although the performance gains over the benchmark settings were relatively modest. Nevertheless, the proposed method consistently outperformed the corresponding benchmark configurations, indicating that the proposed feature-fusion strategy remains effective even under the more challenging subject-independent evaluation protocol.

Overall, the LOSO results obtained on the KDEF dataset are consistent with those observed on the JAFFE dataset, further demonstrating that the proposed feature-fusion strategy effectively improves subject-independent facial emotion recognition across different CNN backbones.

Table 14 summarizes the computational efficiency of the benchmark methods and the proposed method in terms of training and inference time under LOSO cross-validation on the KDEF dataset. Consistent with the observations on the JAFFE dataset under LOSO evaluation, incorporating additional handcrafted features gradually increased both the training and inference time for all evaluated backbone networks because of the additional computation required for handcrafted feature extraction and feature fusion. Nevertheless, the additional computational overhead remained modest relative to the corresponding improvement in subject-independent recognition performance.

Among the evaluated backbone networks, LeNet-5 again required the shortest training and inference time owing to its lightweight architecture, whereas ResNet-18 exhibited the highest computational cost because of its substantially larger network capacity. MobileNetV2 maintained considerably lower computational cost than ResNet-18 while preserving competitive subject-independent recognition performance.

Overall, the computational efficiency results obtained under LOSO cross-validation are consistent with those observed under five-fold cross-validation, demonstrating that the proposed feature-fusion strategy maintains a favorable balance between subject-independent generalization capability and computational efficiency across different CNN backbones.

#### 6.2.2. Statistical Analysis

Paired *t*-tests were performed to evaluate the statistical significance of the observed performance differences between the benchmark methods and the proposed method. The complete statistical analysis results for each backbone network and evaluation protocol are provided in the Supplementary Materials (Section S5.2).

## 7. Results on the Official RAF-DB Train/Test Split

### 7.1. Ablation Study

Table 15 presents the quantitative performance comparison of the benchmark methods and the proposed method on the official RAF-DB train/test split using three different CNN backbones. Compared with the controlled datasets evaluated in the previous sections, RAF-DB provides a substantially more challenging in-the-wild evaluation scenario because of its large variations in facial pose, illumination, occlusion, and spontaneous facial expressions.

Despite the increased difficulty of the dataset, the proposed method consistently achieved the best overall recognition performance across all evaluated backbone networks. For the LeNet-5 backbone, the proposed method improved the accuracy from 0.7056 for the CNN-only baseline (Bench. 1) to 0.7420. Similar improvements were observed for the ResNet-18 and MobileNetV2 backbones, where the proposed method achieved the highest macro-average TPR, PPV, F1-score, and overall accuracy among the evaluated feature settings.

These results demonstrate that the proposed angular and HOG features continue to provide complementary information under realistic facial emotion recognition conditions. Although the overall recognition performance was lower than that obtained on the controlled datasets, the proposed feature-fusion strategy consistently improved recognition performance across all evaluated backbone networks.

Overall, the experimental results on the official RAF-DB train/test split demonstrate that the proposed feature-fusion strategy generalizes effectively from controlled laboratory datasets to more challenging real-world facial emotion recognition scenarios.

To provide a more detailed class-wise analysis, the complete per-class performance results for all backbone networks are provided in the Supplementary Materials (Section S6).

Table 16 summarizes the computational efficiency of the benchmark methods and the proposed method in terms of training and inference time on the official RAF-DB train/test split. Consistent with the observations on the controlled datasets, incorporating additional handcrafted features gradually increased both the training and inference time for all evaluated backbone networks because of the additional computation required for handcrafted feature extraction and feature fusion. Although the overall training time increased owing to the substantially larger size of the RAF-DB dataset, the additional computational overhead introduced by the proposed method remained modest relative to the corresponding improvement in recognition performance.

Among the evaluated backbone networks, LeNet-5 again required the shortest training and inference time because of its lightweight architecture, whereas ResNet-18 exhibited the highest computational cost because of its substantially larger network capacity. MobileNetV2 required considerably less training time than ResNet-18 while maintaining competitive recognition performance, demonstrating the computational efficiency of lightweight CNN architectures under realistic in-the-wild evaluation conditions.

Overall, the computational efficiency results on the RAF-DB dataset are consistent with those obtained on the controlled datasets, indicating that the proposed feature-fusion strategy maintains a favorable balance between recognition performance and computational cost even for large-scale real-world facial emotion recognition tasks.

### 7.2. Comparison with Existing Methods

Table 17 presents the performance comparison of the proposed method with representative facial emotion recognition approaches on the official RAF-DB train/test split, including conventional handcrafted feature-based methods (CH), standard CNN models (CNN), transfer learning-based models (TL), and hybrid feature-fusion methods (HF).

Despite the increased complexity of the RAF-DB dataset, the proposed method consistently achieved competitive or superior recognition performance across all benchmark categories. Compared with the CH methods and representative CNN models, the proposed method substantially improved the overall recognition performance, demonstrating that the proposed combination of angular and HOG features effectively complements CNN-based deep representations under realistic in-the-wild conditions. In particular, the proposed method using the LeNet-5 backbone achieved the highest overall accuracy of 0.7420, while the proposed MobileNetV2 backbone also achieved competitive recognition performance despite its lightweight architecture.

The proposed method also outperformed the representative transfer learning-based and hybrid feature-fusion approaches. In particular, the proposed LeNet-5 backbone achieved higher overall recognition performance than VGG-19, demonstrating that carefully designed handcrafted geometric and local texture features can effectively compensate for the limited representation capability of lightweight CNN architectures. Similar to the observations on the controlled datasets, the relatively lower performance of ResNet-152 is likely related to its substantially larger model capacity, which makes optimization more challenging despite transfer learning.

Overall, these comparative results demonstrate that the proposed feature-fusion strategy generalizes effectively from controlled laboratory datasets to realistic in-the-wild facial emotion recognition scenarios while maintaining competitive performance across diverse benchmark categories.

## 8. Results on the Official AffectNet Train/Test Split

### 8.1. Ablation Study

Table 18 presents the quantitative performance comparison of the benchmark methods and the proposed method on the official AffectNet train/test split using three different CNN backbones. AffectNet is one of the largest and most challenging in-the-wild facial emotion recognition datasets, containing substantial variations in pose, illumination, occlusion, facial appearance, and spontaneous emotional expressions.

Despite the increased complexity of the dataset, the proposed method consistently achieved the highest overall recognition performance across all evaluated backbone networks. For the LeNet-5 backbone, the proposed method improved the accuracy from 0.4460 for the CNN-only baseline (Bench. 1) to 0.5250. Similar improvements were observed for the ResNet-18 and MobileNetV2 backbones, where the proposed method consistently achieved the highest macro-average TPR, PPV, F1-score, and overall accuracy among the evaluated feature settings.

Compared with the controlled datasets and the RAF-DB dataset, the overall recognition performance on AffectNet was relatively lower because of the substantially greater diversity and complexity of real-world facial expressions. Nevertheless, the proposed feature-fusion strategy consistently improved recognition performance across all evaluated backbone networks, demonstrating that the proposed angular and HOG features remain effective even under highly challenging large-scale in-the-wild conditions.

Overall, the experimental results on AffectNet provide strong evidence that the proposed feature-fusion strategy generalizes effectively from controlled laboratory datasets to large-scale real-world facial emotion recognition scenarios while maintaining consistent performance improvements across different CNN backbones.

To provide a more detailed class-wise analysis, the complete per-class performance results for all backbone networks are provided in the Supplementary Materials (Section S7).

Table 19 summarizes the computational efficiency of the benchmark methods and the proposed method in terms of training and inference time on the official AffectNet train/test split. Consistent with the observations on the controlled datasets and the RAF-DB dataset, incorporating additional handcrafted features gradually increased both the training and inference time for all evaluated backbone networks because of the additional computation required for handcrafted feature extraction and feature fusion. Although the overall training time increased owing to the substantially larger size and diversity of the AffectNet dataset, the additional computational overhead introduced by the proposed method remained modest relative to the corresponding improvement in recognition performance.

Among the evaluated backbone networks, LeNet-5 again required the shortest training and inference time owing to its lightweight architecture, whereas ResNet-18 exhibited the highest computational cost because of its substantially larger network capacity. MobileNetV2 maintained considerably lower computational cost than ResNet-18 while providing competitive recognition performance, demonstrating the computational efficiency of lightweight CNN architectures for large-scale real-world facial emotion recognition.

Overall, the computational efficiency results on AffectNet are consistent with those obtained on the controlled datasets and the RAF-DB dataset, demonstrating that the proposed feature-fusion strategy maintains a favorable balance between recognition performance and computational efficiency even under highly challenging large-scale in-the-wild conditions.

### 8.2. Comparison with Existing Methods

Table 20 presents the performance comparison of the proposed method with representative facial emotion recognition approaches on the official AffectNet train/test split, including conventional handcrafted feature-based methods (CH), standard CNN models (CNN), transfer learning-based models (TL), and hybrid feature-fusion methods (HF).

Despite the substantial complexity of the AffectNet dataset, the proposed method consistently achieved superior recognition performance across all benchmark categories. Compared with the CH methods and representative CNN models, the proposed method substantially improved all evaluation metrics, demonstrating that the proposed combination of angular and HOG features effectively complements CNN-based deep representations under highly challenging real-world conditions. In particular, the proposed method using the ResNet-18 backbone achieved the highest overall recognition performance, while the proposed LeNet-5 and MobileNetV2 backbones also consistently outperformed the corresponding baseline CNN models.

The proposed method also demonstrated competitive or superior performance compared with representative transfer learning-based and hybrid feature-fusion approaches. Notably, the proposed method using the lightweight LeNet-5 backbone achieved higher overall recognition performance than VGG-19, suggesting that carefully designed handcrafted geometric and local texture features can effectively compensate for the relatively limited representation capability of lightweight CNN architectures. Similar to the observations on the previous datasets, the relatively lower performance of ResNet-152 is likely related to its substantially larger model capacity, which increases the optimization difficulty under the highly diverse AffectNet dataset.

Overall, the comparative results on AffectNet provide strong evidence that the proposed feature-fusion strategy generalizes effectively to large-scale in-the-wild facial emotion recognition scenarios while maintaining competitive performance across diverse benchmark categories and CNN backbones.

## 9. Discussion

The experimental results consistently demonstrate that combining CNN-based deep features with handcrafted geometric and local texture features improves facial emotion recognition performance across multiple evaluation protocols and datasets. Unlike deep representations, which primarily capture high-level semantic information, the proposed angular and HOG features explicitly encode localized geometric deformations and contour characteristics associated with facial expressions. Consequently, the proposed feature-fusion strategy provides complementary information that cannot be fully captured by deep representations alone, thereby resulting in more discriminative facial emotion representations.

The Grad-CAM analysis further provides a rationale for the proposed handcrafted feature design. The activation maps consistently indicate that the CNN primarily focuses on the central and lower facial regions, particularly around the nose and mouth. This observation consistently supports the decision to extract HOG features from the nose and mouth regions rather than from the entire face across all three controlled datasets. In addition, the proposed angular features explicitly describe geometric variations occurring in facial components that are known to change during emotional expressions, including the eyebrows, eyes, nose, and mouth. Together, these observations demonstrate that the handcrafted features complement the attention characteristics of CNN-based deep representations.

Another important finding is that the proposed feature-fusion strategy consistently improved recognition performance under substantially different evaluation scenarios. Similar performance improvements were observed not only on controlled laboratory datasets (JAFFE, CK+, and KDEF) but also under subject-independent evaluation using leave-one-subject-out cross-validation and on large-scale in-the-wild datasets (RAF-DB and AffectNet). These results suggest that the proposed handcrafted features generalize well across different datasets, subject populations, and real-world imaging conditions, as further evidenced by the consistent improvements obtained on the challenging RAF-DB and AffectNet datasets.

Another noteworthy observation is that the proposed feature-fusion strategy consistently improved facial emotion recognition performance across different CNN backbone networks, including LeNet-5, ResNet-18, and MobileNetV2. Although the magnitude of improvement varied according to the representation capability of each backbone, the proposed handcrafted features consistently provided complementary information beyond the learned deep representations. Interestingly, the largest performance gains were observed for the lightweight LeNet-5 backbone, indicating that handcrafted geometric and local texture features effectively compensate for the limited representational capacity of shallow CNN models. This observation is consistent with recent studies demonstrating the effectiveness of transfer learning for facial expression recognition using deep CNN backbone networks [39]. While transfer learning enables CNN models to exploit rich deep representations learned from large-scale datasets, the present work demonstrates that handcrafted geometric and local texture features provide additional complementary information that consistently improves recognition performance across different backbone architectures.

The statistical analyses further support these observations. For the lightweight LeNet-5 backbone, statistically significant performance improvements were consistently observed across multiple evaluation metrics, whereas the improvements obtained with the deeper backbone networks were generally smaller. This observation suggests that handcrafted features become particularly valuable when the representational capacity of the CNN backbone is relatively limited.

The computational efficiency analysis demonstrated that the proposed feature-fusion strategy introduces only a modest increase in training and inference time compared with the CNN-only baselines. Considering the consistent performance improvements obtained across multiple datasets and evaluation protocols, the additional computational cost represents a favorable trade-off between recognition accuracy and computational efficiency, particularly for practical facial emotion recognition systems deployed on resource-constrained platforms.

Beyond the technical performance of the proposed method, the ethical and societal implications of facial emotion recognition, as a sensitive affective computing technology, should also be carefully considered. Although this study used only publicly available datasets in accordance with their respective licensing conditions, facial emotion recognition inherently involves the analysis of facial images, which may raise concerns regarding privacy and responsible data usage in practical applications. Misclassification may lead to inappropriate decisions or interventions if facial emotion recognition is used without considering additional contextual information. Therefore, the proposed method should be regarded as an assistive tool rather than a fully autonomous decision-making system.

In addition, the datasets adopted in this study may not fully represent the diversity of real-world populations with respect to age, ethnicity, gender, and cultural background. Consequently, potential demographic bias may influence the recognition performance for specific population groups. Future studies should therefore investigate the fairness, demographic bias, and robustness of the proposed method using more diverse facial emotion recognition datasets.

Furthermore, facial expressions alone cannot always reliably represent an individual’s true emotional state because emotional expressions are often influenced by personal, cultural, and contextual factors. Therefore, the recognition results produced by facial emotion recognition systems should not be interpreted as definitive evidence of a person’s emotional condition. This consideration is particularly important in sensitive application domains such as healthcare and education, where facial emotion recognition should be used together with contextual information and appropriate human oversight.

Despite these promising results, several limitations remain. First, the proposed method assumes that major facial components are clearly visible because both the angular features and HOG features rely on accurate facial landmark localization. Consequently, severe occlusions, extreme head poses, or inaccurate landmark detection may reduce recognition performance. Second, although the proposed feature-fusion strategy demonstrated consistent improvements across multiple datasets and evaluation protocols, the current implementation relies on simple feature-level concatenation and has not yet been validated on more demographically diverse populations. More sophisticated adaptive fusion strategies and broader demographic evaluations may further improve both robustness and fairness.

Future work will focus on developing more robust feature-fusion strategies capable of handling severe occlusions, large pose variations, and challenging real-world imaging conditions. In addition, attention-based or transformer-based fusion mechanisms, multimodal affective computing systems, video-based facial emotion recognition, and fairness-aware learning using more demographically diverse datasets will be investigated.

Overall, the experimental findings demonstrate that integrating handcrafted geometric and local texture features with CNN-based deep representations provides an effective and computationally efficient framework for facial emotion recognition across diverse evaluation scenarios.

## 10. Conclusions

This study presented a hybrid facial emotion recognition framework that integrates CNN-based deep features with handcrafted geometric and local texture features. The proposed method was comprehensively evaluated using three controlled datasets (JAFFE, CK+, and KDEF), two large-scale in-the-wild datasets (RAF-DB and AffectNet), five-fold cross-validation, leave-one-subject-out cross-validation, statistical significance analysis, and computational efficiency evaluation. Experimental results consistently demonstrated that the proposed feature-fusion strategy improved facial emotion recognition performance across different datasets, evaluation protocols, and CNN backbone networks while maintaining a favorable balance between recognition performance and computational efficiency. These findings demonstrate that handcrafted geometric and local texture features effectively complement CNN-based deep representations, enabling robust facial emotion recognition across both controlled and large-scale in-the-wild evaluation scenarios. Future work will focus on developing more advanced feature-fusion strategies, improving robustness to occlusions and pose variations, investigating fairness across diverse demographic populations, and extending the proposed framework to multimodal and video-based facial emotion recognition.

## Figures and Tables

**Table 1:** Feature combinations used in the benchmark methods and the proposed method.

Setting	CNN Feature	Angle	HOG
Bench. 1	🗸	–	–
Bench. 2	🗸	🗸	–
Bench. 3	🗸	–	🗸
Proposed	🗸	🗸	🗸

*Note:* 🗸 = feature included; “–” = feature not included.

**Table 2:** Quantitative performance comparison of the benchmark methods (Bench. 1–3) and the proposed method under five-fold cross-validation on the JAFFE dataset.

Backbone	Setting	Macro-Average			ACC
		TPR	PPV	F1	
LeNet-5	Bench. 1	0.678 ± 0.009	0.683 ± 0.013	0.749 ± 0.037	0.683 ± 0.019
	Bench. 2	0.754 ± 0.061	0.756 ± 0.062	0.785 ± 0.055	0.748 ± 0.063
	Bench. 3	0.765 ± 0.039	0.765 ± 0.040	0.782 ± 0.050	0.757 ± 0.047
	Proposed	0.766 ± 0.037	0.767 ± 0.030	0.791 ± 0.024	0.758 ± 0.041
ResNet-18	Bench. 1	0.681 ± 0.087	0.670 ± 0.096	0.717 ± 0.090	0.703 ± 0.085
	Bench. 2	0.725 ± 0.043	0.693 ± 0.045	0.720 ± 0.045	0.712 ± 0.046
	Bench. 3	0.733 ± 0.042	0.767 ± 0.041	0.721 ± 0.041	0.719 ± 0.043
	Proposed	0.766 ± 0.033	0.778 ± 0.035	0.770 ± 0.030	0.762 ± 0.025
MobileNetV2	Bench. 1	0.672 ± 0.076	0.673 ± 0.076	0.713 ± 0.057	0.673 ± 0.069
	Bench. 2	0.716 ± 0.076	0.718 ± 0.078	0.724 ± 0.075	0.711 ± 0.082
	Bench. 3	0.688 ± 0.049	0.688 ± 0.051	0.717 ± 0.046	0.680 ± 0.056
	Proposed	0.726 ± 0.074	0.726 ± 0.076	0.749 ± 0.072	0.722 ± 0.080

**Table 3:** Training and inference time of the benchmark methods (Bench. 1–3) and the proposed method under five-fold cross-validation on the JAFFE dataset.

Backbone	Setting	Training Time (s)	Inference Time (ms/Image)
LeNet-5	Bench. 1	15.26 ± 0.67	0.065 ± 0.010
	Bench. 2	16.59 ± 0.67	0.073 ± 0.007
	Bench. 3	18.94 ± 0.17	0.076 ± 0.007
	Proposed	19.96 ± 0.21	0.086 ± 0.011
ResNet-18	Bench. 1	502.67 ± 26.76	0.069 ± 0.006
	Bench. 2	528.83 ± 29.98	0.078 ± 0.019
	Bench. 3	553.61 ± 34.26	0.083 ± 0.010
	Proposed	556.26 ± 38.39	0.087 ± 0.012
MobileNetV2	Bench. 1	337.18 ± 21.93	0.065 ± 0.003
	Bench. 2	341.07 ± 17.41	0.073 ± 0.006
	Bench. 3	350.90 ± 18.22	0.076 ± 0.008
	Proposed	362.97 ± 17.25	0.076 ± 0.009

**Table 4:** Comparison of the proposed method with representative facial emotion recognition methods under five-fold cross-validation on the JAFFE dataset.

Category	Reference/ Model	Macro-Average			ACC
		TPR	PPV	F1	
CH	[16]	0.545 ± 0.079	0.580 ± 0.126	0.504 ± 0.112	0.496 ± 0.158
	[17]	0.637 ± 0.027	0.571 ± 0.101	0.536 ± 0.145	0.580 ± 0.040
CNN	LeNet-5	0.504 ± 0.134	0.508 ± 0.139	0.521 ± 0.141	0.492 ± 0.147
	AlexNet	0.492 ± 0.137	0.493 ± 0.137	0.460 ± 0.206	0.449 ± 0.177
	ResNet-18	0.738 ± 0.026	0.740 ± 0.027	0.782 ± 0.033	0.737 ± 0.023
TL	VGG-19	0.732 ± 0.046	0.737 ± 0.048	0.800 ± 0.028	0.729 ± 0.052
	ResNet-152	0.438 ± 0.084	0.435 ± 0.084	0.464 ± 0.132	0.423 ± 0.095
HF	[37]	0.597 ± 0.066	0.572 ± 0.093	0.595 ± 0.059	0.625 ± 0.021
	[38]	0.679 ± 0.089	0.616 ± 0.085	0.629 ± 0.120	0.514 ± 0.068
Proposed	LeNet-5	0.766 ± 0.037	0.767 ± 0.030	0.791 ± 0.024	0.758 ± 0.041
	ResNet-18	0.766 ± 0.033	0.778 ± 0.035	0.770 ± 0.030	0.762 ± 0.025
	MobileNetV2	0.726 ± 0.074	0.726 ± 0.076	0.749 ± 0.072	0.722 ± 0.080

**Table 5:** Quantitative performance comparison of the benchmark methods (Bench. 1–3) and the proposed method under five-fold cross-validation on the CK+ dataset.

Backbone	Setting	Macro-Average			ACC
		TPR	PPV	F1	
LeNet-5	Bench. 1	0.588 ± 0.028	0.586 ± 0.015	0.567 ± 0.017	0.655 ± 0.015
	Bench. 2	0.668 ± 0.043	0.684 ± 0.040	0.656 ± 0.042	0.749 ± 0.036
	Bench. 3	0.682 ± 0.055	0.699 ± 0.095	0.672 ± 0.076	0.758 ± 0.052
	Proposed	0.750 ± 0.050	0.773 ± 0.057	0.750 ± 0.052	0.823 ± 0.028
ResNet-18	Bench. 1	0.614 ± 0.037	0.640 ± 0.024	0.575 ± 0.058	0.618 ± 0.068
	Bench. 2	0.609 ± 0.081	0.591 ± 0.086	0.665 ± 0.074	0.655 ± 0.089
	Bench. 3	0.593 ± 0.046	0.726 ± 0.020	0.695 ± 0.024	0.695 ± 0.072
	Proposed	0.637 ± 0.045	0.753 ± 0.058	0.805 ± 0.025	0.813 ± 0.046
MobileNetV2	Bench. 1	0.599 ± 0.016	0.647 ± 0.069	0.591 ± 0.010	0.731 ± 0.023
	Bench. 2	0.564 ± 0.048	0.557 ± 0.050	0.543 ± 0.043	0.713 ± 0.025
	Bench. 3	0.594 ± 0.041	0.613 ± 0.048	0.585 ± 0.037	0.731 ± 0.026
	Proposed	0.616 ± 0.044	0.612 ± 0.070	0.603 ± 0.051	0.740 ± 0.032

**Table 6:** Training and inference time of the benchmark methods (Bench. 1–3) and the proposed method under five-fold cross-validation on the CK+ dataset.

Backbone	Setting	Training Time (s)	Inference Time (ms/Image)
LeNet-5	Bench. 1	14.88 ± 0.24	0.058 ± 0.004
	Bench. 2	16.16 ± 0.20	0.068 ± 0.010
	Bench. 3	17.72 ± 0.16	0.070 ± 0.007
	Proposed	19.72 ± 0.15	0.075 ± 0.011
ResNet-18	Bench. 1	604.04 ± 37.67	0.066 ± 0.010
	Bench. 2	632.31 ± 39.94	0.076 ± 0.018
	Bench. 3	660.56 ± 41.13	0.080 ± 0.011
	Proposed	688.11 ± 42.34	0.094 ± 0.031
MobileNetV2	Bench. 1	252.60 ± 28.12	0.063 ± 0.007
	Bench. 2	263.75 ± 30.08	0.068 ± 0.013
	Bench. 3	270.68 ± 30.13	0.073 ± 0.020
	Proposed	284.66 ± 29.14	0.083 ± 0.018

**Table 7:** Comparison of the proposed method with representative facial emotion recognition methods under five-fold cross-validation on the CK+ dataset.

Category	Reference/ Model	Macro-Average			ACC
		TPR	PPV	F1	
CH	[16]	0.430 ± 0.082	0.613 ± 0.080	0.517 ± 0.135	0.633 ± 0.091
	[17]	0.501 ± 0.129	0.433 ± 0.062	0.433 ± 0.052	0.598 ± 0.114
CNN	LeNet-5	0.531 ± 0.090	0.543 ± 0.109	0.520 ± 0.097	0.655 ± 0.066
	AlexNet	0.542 ± 0.079	0.563 ± 0.107	0.527 ± 0.083	0.658 ± 0.066
	ResNet-18	0.593 ± 0.035	0.656 ± 0.050	0.583 ± 0.022	0.700 ± 0.039
TL	VGG-19	0.646 ± 0.103	0.669 ± 0.111	0.638 ± 0.101	0.740 ± 0.066
	ResNet-152	0.434 ± 0.064	0.502 ± 0.072	0.428 ± 0.090	0.575 ± 0.060
HF	[37]	0.439 ± 0.069	0.738 ± 0.123	0.607 ± 0.056	0.644 ± 0.088
	[38]	0.726 ± 0.107	0.445 ± 0.061	0.593 ± 0.067	0.550 ± 0.052
Proposed	LeNet-5	0.750 ± 0.050	0.773 ± 0.057	0.750 ± 0.052	0.823 ± 0.028
	ResNet-18	0.637 ± 0.045	0.753 ± 0.058	0.805 ± 0.025	0.813 ± 0.046
	MobileNetV2	0.616 ± 0.044	0.612 ± 0.070	0.603 ± 0.051	0.740 ± 0.032

**Table 8:** Quantitative performance comparison of the benchmark methods (Bench. 1–3) and the proposed method under five-fold cross-validation on the KDEF dataset.

Backbone	Setting	Macro-Average			ACC
		TPR	PPV	F1	
LeNet-5	Bench. 1	0.702 ± 0.025	0.702 ± 0.022	0.698 ± 0.024	0.702 ± 0.025
	Bench. 2	0.796 ± 0.019	0.799 ± 0.017	0.794 ± 0.018	0.796 ± 0.019
	Bench. 3	0.821 ± 0.026	0.825 ± 0.028	0.819 ± 0.027	0.821 ± 0.026
	Proposed	0.843 ± 0.023	0.850 ± 0.024	0.842 ± 0.023	0.843 ± 0.023
ResNet-18	Bench. 1	0.734 ± 0.035	0.746 ± 0.030	0.719 ± 0.041	0.710 ± 0.036
	Bench. 2	0.796 ± 0.020	0.821 ± 0.032	0.804 ± 0.021	0.709 ± 0.028
	Bench. 3	0.840 ± 0.029	0.827 ± 0.018	0.776 ± 0.026	0.761 ± 0.035
	Proposed	0.846 ± 0.029	0.846 ± 0.030	0.825 ± 0.032	0.821 ± 0.028
MobileNetV2	Bench. 1	0.782 ± 0.026	0.780 ± 0.028	0.778 ± 0.028	0.782 ± 0.026
	Bench. 2	0.787 ± 0.031	0.791 ± 0.033	0.784 ± 0.037	0.787 ± 0.031
	Bench. 3	0.805 ± 0.029	0.806 ± 0.030	0.802 ± 0.030	0.805 ± 0.029
	Proposed	0.812 ± 0.042	0.813 ± 0.044	0.808 ± 0.044	0.812 ± 0.042

**Table 9:** Training and inference time of the benchmark methods (Bench. 1–3) and the proposed method under five-fold cross-validation on the KDEF dataset.

Backbone	Setting	Training Time (s)	Inference Time (ms/Image)
LeNet-5	Bench. 1	15.20 ± 1.60	0.056 ± 0.006
	Bench. 2	17.41 ± 1.27	0.065 ± 0.008
	Bench. 3	18.71 ± 1.53	0.068 ± 0.007
	Proposed	19.65 ± 1.41	0.078 ± 0.011
ResNet-18	Bench. 1	612.39 ± 30.90	0.074 ± 0.007
	Bench. 2	633.75 ± 35.28	0.085 ± 0.005
	Bench. 3	648.16 ± 37.09	0.089 ± 0.004
	Proposed	658.41 ± 36.30	0.098 ± 0.003
MobileNetV2	Bench. 1	226.03 ± 34.92	0.069 ± 0.009
	Bench. 2	231.84 ± 35.18	0.075 ± 0.007
	Bench. 3	238.86 ± 33.22	0.079 ± 0.002
	Proposed	245.74 ± 34.11	0.088 ± 0.005

**Table 10:** Comparison of the proposed method with representative facial emotion recognition methods under five-fold cross-validation on the KDEF dataset.

Category	Reference/ Model	Macro-Average			ACC
		TPR	PPV	F1	
CH	[16]	0.626 ± 0.033	0.688 ± 0.033	0.713 ± 0.026	0.750 ± 0.025
	[17]	0.695 ± 0.039	0.658 ± 0.016	0.723 ± 0.011	0.767 ± 0.010
CNN	LeNet-5	0.699 ± 0.044	0.702 ± 0.040	0.694 ± 0.045	0.702 ± 0.040
	AlexNet	0.733 ± 0.012	0.726 ± 0.006	0.710 ± 0.005	0.726 ± 0.006
	ResNet-18	0.768 ± 0.028	0.745 ± 0.033	0.743 ± 0.031	0.745 ± 0.033
TL	VGG-19	0.780 ± 0.025	0.763 ± 0.026	0.760 ± 0.027	0.763 ± 0.026
	ResNet-152	0.601 ± 0.015	0.599 ± 0.017	0.596 ± 0.015	0.599 ± 0.017
HF	[37]	0.734 ± 0.027	0.749 ± 0.015	0.714 ± 0.046	0.804 ± 0.012
	[38]	0.707 ± 0.021	0.654 ± 0.027	0.734 ± 0.030	0.710 ± 0.021
Proposed	LeNet-5	0.843 ± 0.023	0.850 ± 0.024	0.842 ± 0.023	0.843 ± 0.023
	ResNet-18	0.846 ± 0.029	0.846 ± 0.030	0.825 ± 0.032	0.821 ± 0.028
	MobileNetV2	0.812 ± 0.042	0.813 ± 0.044	0.808 ± 0.044	0.812 ± 0.042

**Table 11:** Quantitative performance comparison of the benchmark methods (Bench. 1–3) and the proposed method under leave-one-subject-out cross-validation on the JAFFE dataset.

Backbone	Setting	Macro-Average			ACC
		TPR	PPV	F1	
LeNet-5	Bench. 1	0.656 ± 0.144	0.571 ± 0.177	0.579 ± 0.166	0.652 ± 0.142
	Bench. 2	0.768 ± 0.174	0.676 ± 0.188	0.682 ± 0.179	0.768 ± 0.175
	Bench. 3	0.739 ± 0.151	0.659 ± 0.161	0.654 ± 0.161	0.740 ± 0.149
	Proposed	0.808 ± 0.168	0.737 ± 0.130	0.736 ± 0.188	0.810 ± 0.170
ResNet-18	Bench. 1	0.740 ± 0.217	0.655 ± 0.145	0.602 ± 0.211	0.651 ± 0.147
	Bench. 2	0.769 ± 0.157	0.647 ± 0.178	0.652 ± 0.162	0.721 ± 0.146
	Bench. 3	0.774 ± 0.154	0.655 ± 0.143	0.643 ± 0.147	0.711 ± 0.174
	Proposed	0.790 ± 0.173	0.670 ± 0.157	0.687 ± 0.153	0.732 ± 0.115
MobileNetV2	Bench. 1	0.756 ± 0.121	0.687 ± 0.127	0.680 ± 0.124	0.756 ± 0.120
	Bench. 2	0.717 ± 0.121	0.668 ± 0.127	0.657 ± 0.123	0.715 ± 0.120
	Bench. 3	0.721 ± 0.186	0.677 ± 0.121	0.664 ± 0.118	0.719 ± 0.218
	Proposed	0.757 ± 0.135	0.695 ± 0.128	0.694 ± 0.127	0.762 ± 0.124

**Table 12:** Training and inference time of the benchmark methods (Bench. 1–3) and the proposed method under leave-one-subject-out cross-validation on the JAFFE dataset.

Backbone	Setting	Training Time (s)	Inference Time (ms/Image)
LeNet-5	Bench. 1	16.35 ± 0.59	0.064 ± 0.011
	Bench. 2	18.37 ± 0.21	0.078 ± 0.007
	Bench. 3	20.17 ± 0.14	0.079 ± 0.008
	Proposed	21.57 ± 0.15	0.085 ± 0.010
ResNet-18	Bench. 1	526.53 ± 30.47	0.070 ± 0.009
	Bench. 2	539.63 ± 38.61	0.074 ± 0.008
	Bench. 3	560.09 ± 35.72	0.083 ± 0.009
	Proposed	572.36 ± 36.19	0.084 ± 0.003
MobileNetV2	Bench. 1	357.45 ± 21.33	0.065 ± 0.004
	Bench. 2	363.28 ± 18.45	0.075 ± 0.006
	Bench. 3	380.77 ± 20.70	0.077 ± 0.009
	Proposed	388.04 ± 26.14	0.078 ± 0.006

**Table 13:** Quantitative performance comparison of the benchmark methods (Bench. 1–3) and the proposed method under leave-one-subject-out cross-validation on the KDEF dataset.

Backbone	Setting	Macro-Average			ACC
		TPR	PPV	F1	
LeNet-5	Bench. 1	0.666 ± 0.162	0.622 ± 0.209	0.615 ± 0.187	0.666 ± 0.162
	Bench. 2	0.785 ± 0.131	0.750 ± 0.184	0.743 ± 0.162	0.785 ± 0.131
	Bench. 3	0.781 ± 0.135	0.774 ± 0.180	0.750 ± 0.163	0.781 ± 0.135
	Proposed	0.802 ± 0.125	0.797 ± 0.169	0.773 ± 0.152	0.802 ± 0.125
ResNet-18	Bench. 1	0.612 ± 0.197	0.692 ± 0.165	0.644 ± 0.105	0.601 ± 0.108
	Bench. 2	0.718 ± 0.132	0.637 ± 0.187	0.652 ± 0.134	0.618 ± 0.116
	Bench. 3	0.758 ± 0.189	0.695 ± 0.141	0.776 ± 0.124	0.652 ± 0.165
	Proposed	0.779 ± 0.179	0.733 ± 0.112	0.790 ± 0.116	0.770 ± 0.125
MobileNetV2	Bench. 1	0.780 ± 0.140	0.757 ± 0.189	0.748 ± 0.163	0.780 ± 0.140
	Bench. 2	0.783 ± 0.135	0.766 ± 0.182	0.749 ± 0.163	0.783 ± 0.135
	Bench. 3	0.789 ± 0.141	0.772 ± 0.178	0.752 ± 0.169	0.789 ± 0.141
	Proposed	0.795 ± 0.136	0.777 ± 0.179	0.763 ± 0.162	0.795 ± 0.136

**Table 14:** Training and inference time of the benchmark methods (Bench. 1–3) and the proposed method under leave-one-subject-out cross-validation on the KDEF dataset.

Backbone	Setting	Training Time (s)	Inference Time (ms/Image)
LeNet-5	Bench. 1	23.09 ± 3.51	0.055 ± 0.006
	Bench. 2	26.02 ± 4.49	0.066 ± 0.004
	Bench. 3	29.60 ± 4.10	0.071 ± 0.006
	Proposed	32.60 ± 5.10	0.076 ± 0.005
ResNet-18	Bench. 1	652.16 ± 48.61	0.074 ± 0.007
	Bench. 2	662.83 ± 43.33	0.079 ± 0.015
	Bench. 3	675.45 ± 31.48	0.085 ± 0.017
	Proposed	688.51 ± 42.12	0.092 ± 0.016
MobileNetV2	Bench. 1	237.00 ± 36.09	0.067 ± 0.004
	Bench. 2	240.53 ± 40.09	0.074 ± 0.011
	Bench. 3	253.51 ± 39.31	0.078 ± 0.005
	Proposed	260.33 ± 37.11	0.084 ± 0.009

**Table 15:** Quantitative performance comparison of the benchmark methods (Bench. 1–3) and the proposed method on the RAF-DB dataset.

Backbone	Setting	Macro-Average			ACC
		TPR	PPV	F1	
LeNet-5	Bench. 1	0.5550	0.5936	0.5661	0.7056
	Bench. 2	0.5433	0.6586	0.5606	0.7249
	Bench. 3	0.5285	0.6569	0.5467	0.7186
	Proposed	0.5938	0.6658	0.6139	0.7420
ResNet-18	Bench. 1	0.4842	0.5373	0.4892	0.6583
	Bench. 2	0.5290	0.5418	0.4955	0.6693
	Bench. 3	0.4854	0.5666	0.5257	0.6741
	Proposed	0.5676	0.5819	0.5552	0.6961
MobileNetV2	Bench. 1	0.4885	0.5862	0.5196	0.6746
	Bench. 2	0.5414	0.6059	0.5615	0.7098
	Bench. 3	0.5390	0.5850	0.5549	0.7044
	Proposed	0.5717	0.6237	0.5904	0.7307

**Table 16:** Training and inference time of the benchmark methods (Bench. 1–3) and the proposed method on the RAF-DB dataset.

Backbone	Setting	Training Time (s)	Inference Time (ms/Image)
LeNet-5	Bench. 1	95.43	0.025
	Bench. 2	102.97	0.036
	Bench. 3	109.90	0.040
	Proposed	113.31	0.054
ResNet-18	Bench. 1	2612.99	0.153
	Bench. 2	2712.29	0.163
	Bench. 3	2745.16	0.180
	Proposed	2831.96	0.184
MobileNetV2	Bench. 1	1040.64	0.073
	Bench. 2	1173.42	0.086
	Bench. 3	1268.70	0.095
	Proposed	1337.23	0.104

**Table 17:** Comparison of the proposed method with representative facial emotion recognition methods on the official RAF-DB train/test split.

Category	Reference/ Model	Macro-Average			ACC
		TPR	PPV	F1	
CH	[16]	0.4317	0.4093	0.4628	0.6149
	[17]	0.4581	0.4731	0.5205	0.6312
CNN	LeNet-5	0.5550	0.5936	0.5661	0.7056
	AlexNet	0.5368	0.6301	0.5556	0.7245
	ResNet-18	0.5149	0.5726	0.5168	0.6743
TL	VGG-19	0.5489	0.6915	0.5736	0.7108
	ResNet-152	0.3729	0.5269	0.4020	0.6047
HF	[37]	0.5569	0.5560	0.4996	0.6462
	[38]	0.5046	0.5302	0.4970	0.6596
Proposed	LeNet-5	0.5938	0.6569	0.6139	0.7420
	ResNet-18	0.5676	0.5819	0.5552	0.6961
	MobileNetV2	0.5717	0.6237	0.5904	0.7307

**Table 18:** Quantitative performance comparison of the benchmark methods (Bench. 1–3) and the proposed method on the AffectNet dataset.

Backbone	Setting	Macro-Average			ACC
		TPR	PPV	F1	
LeNet-5	Bench. 1	0.4211	0.4367	0.3954	0.4460
	Bench. 2	0.4813	0.4871	0.4711	0.5037
	Bench. 3	0.4604	0.4895	0.4392	0.4788
	Proposed	0.5083	0.5111	0.4983	0.5250
ResNet-18	Bench. 1	0.4012	0.4688	0.5006	0.4834
	Bench. 2	0.5312	0.5022	0.5121	0.5297
	Bench. 3	0.4101	0.4719	0.5035	0.5068
	Proposed	0.5383	0.5376	0.5355	0.5361
MobileNetV2	Bench. 1	0.4399	0.4639	0.4164	0.4629
	Bench. 2	0.4781	0.4817	0.4661	0.4992
	Bench. 3	0.4762	0.4744	0.4662	0.4971
	Proposed	0.4858	0.4895	0.4746	0.5056

**Table 19:** Training and inference time of the benchmark methods (Bench. 1–3) and the proposed method on the AffectNet dataset.

Backbone	Setting	Training Time (s)	Inference Time (ms/Image)
LeNet-5	Bench. 1	96.43	0.030
	Bench. 2	103.92	0.032
	Bench. 3	113.27	0.043
	Proposed	123.83	0.052
ResNet-18	Bench. 1	2170.07	0.085
	Bench. 2	2221.56	0.092
	Bench. 3	2334.71	0.095
	Proposed	2476.72	0.097
MobileNetV2	Bench. 1	1139.62	0.077
	Bench. 2	1273.99	0.084
	Bench. 3	1383.85	0.093
	Proposed	1513.92	0.096

**Table 20:** Comparison of the proposed method with representative facial emotion recognition methods on the official AffectNet train/test split.

Category	Reference/ Model	Macro-Average			ACC
		TPR	PPV	F1	
CH	[16]	0.3288	0.3863	0.3463	0.3649
	[17]	0.3609	0.3589	0.3119	0.3099
CNN	LeNet-5	0.4211	0.4367	0.3954	0.4460
	AlexNet	0.4429	0.4199	0.4350	0.4394
	ResNet-18	0.4526	0.4566	0.4405	0.4709
TL	VGG-19	0.5160	0.4632	0.4451	0.4806
	ResNet-152	0.3615	0.3918	0.3497	0.3745
HF	[37]	0.4618	0.4744	0.3740	0.4622
	[38]	0.4494	0.3966	0.3732	0.4694
Proposed	LeNet-5	0.5083	0.5111	0.4983	0.5250
	ResNet-18	0.5383	0.5376	0.5355	0.5361
	MobileNetV2	0.4858	0.4895	0.4746	0.5056

## Data Availability

The datasets used in this study are publicly available but are subject to their respective licensing terms and access conditions. The JAFFE dataset is available from the official repository (http://www.kasrl.org/jaffe.html, accessed on 14 July 2026) with permission from the dataset providers. The CK+ dataset is available from the official repository (http://www.jeffcohn.net, accessed on 14 July 2026) upon request. The KDEF dataset is available from the official repository (https://kdef.se, accessed on 14 July 2026) upon request. The RAF-DB dataset is available for research purposes through the official website (http://www.whdeng.cn/RAF/model1.html, accessed on 14 July 2026) after registration. The AffectNet dataset is available for research purposes upon request through the official project website (https://mohammadmahoor.com, accessed on 14 July 2026). The authors do not redistribute any of these datasets because they are subject to their respective licensing and usage restrictions.

## Supplementary Materials

The following supporting information can be downloaded at https://www.mdpi.com/article/10.3390/s26144522/s1.
