# Supplementary material for: Facial Emotion Recognition via Fusion of Deep and Handcrafted Features
Source: Sensors (Basel). 2026 Jul 16;26(14):4522. doi: 10.3390/s26144522 (PMC13416614; doi:10.3390/s26144522)
Supplement: Supplementary file 1 [file sensors-26-04522-s001.zip › sensors-4346299-supplementary.pdf]

Supplementary Materials: Facial Emotion Recognition via Fusion of Deep and Handcrafted Features

Seo Eun Cha [1] and Beom Kwon [2,*]

S1. Grad-CAM Analysis for HOG Region Selection

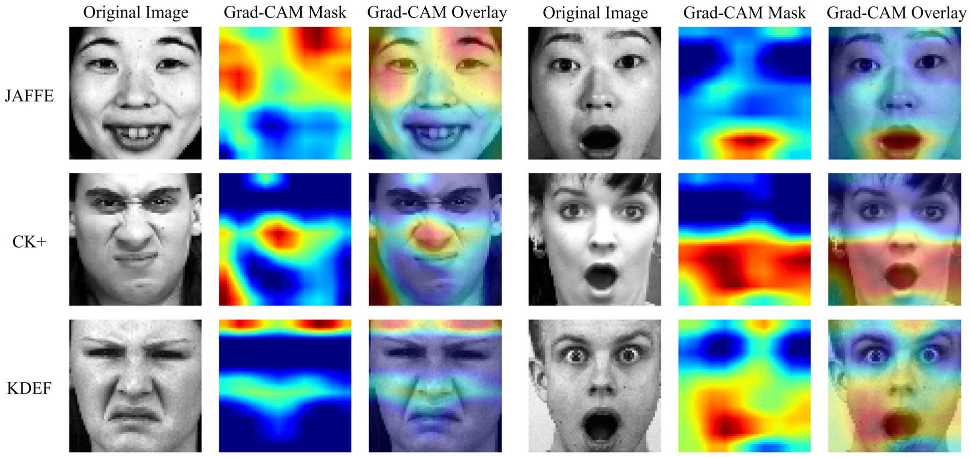

Figure S1. Grad-CAM visualization of the baseline LeNet-5 model for representative correctly classified facial images from the JAFFE, CK+, and KDEF datasets. Across all datasets, the activation maps consistently emphasize the central and lower facial regions, particularly around the nose and mouth, providing empirical support for selecting these regions for HOG feature extraction.

To provide additional evidence supporting the selection of the nose and mouth regions for HOG feature extraction, gradient-weighted class activation mapping (Grad-CAM) analysis was performed using the baseline LeNet-5 model.

For each controlled dataset (JAFFE, CK+, and KDEF), representative facial images correctly classified with a softmax confidence score greater than or equal to 0.85 were selected. Grad-CAM activation maps were subsequently generated to visualize the image regions contributing most strongly to the CNN predictions.

As shown in Figure S1, the CNN consistently focused on the central and lower facial regions, particularly around the nose and mouth, across all three datasets. Only limited activation was observed around the forehead or cheek regions. These observations suggest that local texture variations around the nose and mouth provide highly discriminative information for facial emotion recognition.

The Grad-CAM results therefore provide empirical support for extracting HOG features from the nose and mouth regions rather than from the entire face. This design enables the handcrafted HOG features to complement the deep representations learned by the CNN while avoiding unnecessary background information.

S2. Data Augmentation

This section provides the detailed network architecture and training configuration of the convolutional autoencoder (CAE) used for data augmentation. These implementation details are presented separately from the main manuscript to improve readability while ensuring experimental reproducibility.

To alleviate the limited amount of training data and reduce class imbalance, CAE-based data augmentation was employed. The CAE was applied exclusively to the training

data, whereas the validation and test sets remained completely unchanged throughout all experiments to prevent information leakage. Consequently, no augmented samples were included in the validation or test sets.

Table S1. Detailed network architecture of the convolutional autoencoder (CAE) adopted for training data augmentation.

Layer	Output Size	Configuration
Input	$1 \times 64 \times 64$	Grayscale image
Conv1	$32 \times 64 \times 64$	Kernel size=3 $\times$ 3, stride=1
Conv2	$64 \times 32 \times 32$	Kernel size=3 $\times$ 3, stride=2
Conv3	$128 \times 16 \times 16$	Kernel size=3 $\times$ 3, stride=2
Conv4	$256 \times 8 \times 8$	Kernel size=3 $\times$ 3, stride=2
Conv5	$256 \times 8 \times 8$	Kernel size=3 $\times$ 3, stride=1
Flatten	16,384	
Latent	15	Fully conneted (FC)
FC	16,384	
Deconv1	$256 \times 8 \times 8$	Kernel size=3 $\times$ 3, stride=1
Deconv2	$128 \times 16 \times 16$	Kernel size=3 $\times$ 3, stride=2
Deconv3	$64 \times 32 \times 32$	Kernel size=3 $\times$ 3, stride=2
Deconv4	$32 \times 64 \times 64$	Kernel size=3 $\times$ 3, stride=2
Deconv5	$1 \times 64 \times 64$	Kernel size=3 $\times$ 3, stride=1

The adopted CAE consists of an encoder–decoder architecture. The encoder contains five convolutional layers followed by a fully connected latent representation with a dimensionality of 15, whereas the decoder reconstructs facial images through one fully connected layer and five transposed convolutional layers. Rectified linear unit (ReLU) activation functions were used in the encoder, while a sigmoid activation function was employed in the output layer of the decoder. The detailed architecture of the adopted CAE is summarized in Table S1.

Table S2. Training configuration of the convolutional autoencoder (CAE).

Hyperparameter	Value
Loss	$0.3 \times$ MSE + $0.7 \times$ L1
Optimizer	Adam
Learning rate	0.001
Batch size	8
Epochs	50

The CAE was trained using a weighted reconstruction loss consisting of $0.3\times$ mean squared error (MSE) loss and $0.7\times$ L1 loss. The Adam optimizer was adopted with a learning rate of 0.001, a batch size of 8, and 50 training epochs. The detailed training configuration is summarized in Table S2.

The trained CAE was used to generate additional facial images only for the training set in each experimental split or cross-validation fold. The number of generated images for each emotion category was determined according to the class distribution of the corresponding dataset in order to alleviate class imbalance while preserving the original test distribution.

S3. Implementation Details

This section provides the detailed implementation environment, software versions, training hyperparameters, and reproducibility settings used throughout all experiments. These implementation details are presented separately from the main manuscript to improve readability while ensuring experimental reproducibility.

All experiments were implemented in Python 3.12.13 using PyTorch 2.11.0 with CUDA 12.8. Additional software packages included NumPy 2.0.2, Pandas 2.2.2, OpenCV 4.13.0, Dlib 19.24.6, and Scikit-learn 1.6.1. All experiments were conducted using Google Colab Pro equipped with an NVIDIA Tesla T4 GPU.

Facial regions were detected using the Dlib frontal face detector, and facial landmarks were extracted using the Dlib 68-point facial landmark predictor. HOG features were extracted only from the nose and mouth regions, and PCA was subsequently applied while retaining 90% of the cumulative explained variance.

For all CNN backbone networks, the Adam optimizer was adopted with a learning rate of 0.001, a weight decay of 0.001, and a batch size of 32. The categorical cross-entropy loss function was used for facial emotion classification. During five-fold cross-validation, the training subset was further divided into training and validation sets with a ratio of 8:2. The validation set was used exclusively for early stopping with a patience value of 15.

To ensure experimental reproducibility, all random seeds were fixed to 42, including the Python random seed, NumPy random seed, PyTorch random seed, CUDA random seed, and data partition random state. Furthermore, CuDNN deterministic mode was enabled and benchmark mode was disabled to minimize nondeterministic behavior during model training.

S4. Results under Five-Fold Cross-Validation

S4.1. Results on the JAFFE Dataset

S4.1.1. Per-Class Performance

Table S3. Per-class performance of the benchmark methods (Bench. 1–3) and the proposed method using the LeNet-5 backbone under five-fold cross-validation on the JAFFE dataset.

Setting	Emotion	Macro-Average			ACC
		TPR	PPV	F1	
Bench. 1	AN	0.667±0.167	0.712±0.202	0.679±0.150	0.896±0.049
	DI	0.827±0.119	0.853±0.138	0.830±0.079	0.945±0.027
	FE	0.633±0.223	0.476±0.083	0.526±0.078	0.809±0.028
	HA	0.748±0.170	0.917±0.118	0.804±0.049	0.940±0.012
	SA	0.557±0.290	0.707±0.271	0.552±0.121	0.858±0.042
	SU	0.667±0.167	0.832±0.249	0.709±0.140	0.908±0.059
Bench. 2	AN	0.833±0.118	0.786±0.053	0.804±0.054	0.934±0.015
	DI	0.900±0.091	0.848±0.102	0.869±0.066	0.956±0.025
	FE	0.748±0.190	0.589±0.128	0.653±0.134	0.863±0.051
	HA	0.833±0.167	0.847±0.166	0.822±0.108	0.940±0.041
	SA	0.457±0.192	0.781±0.204	0.550±0.158	0.880±0.037
	SU	0.767±0.190	0.857±0.202	0.792±0.162	0.935±0.053
Bench. 3	AN	0.767±0.091	0.781±0.166	0.772±0.124	0.923±0.046
	DI	0.760±0.146	0.835±0.168	0.775±0.089	0.929±0.032
	FE	0.776±0.219	0.677±0.120	0.711±0.131	0.896±0.036
	HA	0.905±0.147	0.836±0.120	0.862±0.106	0.951±0.036
	SA	0.529±0.282	0.673±0.193	0.544±0.197	0.869±0.035
	SU	0.800±0.139	0.865±0.078	0.823±0.071	0.945±0.019
Proposed	AN	0.700±0.183	0.766±0.180	0.722±0.149	0.913±0.049
	DI	0.827±0.119	0.860±0.135	0.830±0.047	0.946±0.019
	FE	0.776±0.219	0.771±0.159	0.746±0.103	0.912±0.031
	HA	0.933±0.149	0.800±0.163	0.855±0.140	0.945±0.051
	SA	0.552±0.199	0.709±0.176	0.584±0.084	0.874±0.015
	SU	0.867±0.139	0.865±0.078	0.863±0.103	0.956±0.032

Table S3 presents the per-class recognition performance of the benchmark methods and the proposed method using the LeNet-5 backbone under five-fold cross-validation on the JAFFE dataset. The class-wise evaluation provides a more detailed analysis of how the proposed handcrafted features affect the recognition of individual emotion categories.

Compared with the CNN-only baseline (Bench. 1), the proposed method improved the recognition performance for most emotion classes. In particular, noticeable improvements were observed for fear (FE) and surprise (SU), where the proposed method achieved the highest class-wise F1-scores among all feature settings. The F1-score for FE increased from 0.526 ± 0.078 in Bench. 1 to 0.746 ± 0.103 in the proposed method, while the F1-score for SU improved from 0.709 ± 0.140 to 0.863 ± 0.103. These results indicate that the proposed combination of angular and HOG features effectively complements CNN-based deep features for emotion classes characterized by subtle local geometric and texture variations.

The proposed method also maintained competitive performance for the remaining emotion classes while improving the overall balance across different emotions. Although the improvements for some categories, such as disgust (DI) and sadness (SA), were relatively modest, no substantial performance degradation was observed for any emotion class. This observation suggests that the proposed feature-fusion strategy consistently enhances the discriminative capability of the CNN model without sacrificing the recognition performance of other emotion categories.

Overall, the class-wise results are consistent with the macro-average results presented in Table 2, further confirming that the proposed handcrafted features improve facial emotion recognition in a balanced manner across multiple emotion categories rather than benefiting only a small subset of classes.

Table S4. Per-class performance of the benchmark methods (Bench. 1–3) and the proposed method using the ResNet-18 backbone under five-fold cross-validation on the JAFFE dataset.

Setting	Emotion	Macro-Average			ACC
		TPR	PPV	F1	
Bench. 1	AN	0.546±0.121	0.539±0.151	0.669±0.239	0.646±0.075
	DI	0.628±0.118	0.778±0.204	0.723±0.169	0.850±0.037
	FE	0.665±0.107	0.724±0.229	0.723±0.141	0.736±0.048
	HA	0.750±0.143	0.822±0.130	0.769±0.095	0.835±0.033
	SA	0.556±0.072	0.602±0.254	0.615±0.141	0.680±0.018
	SU	0.757±0.238	0.827±0.168	0.889±0.097	0.920±0.088
Bench. 2	AN	0.790±0.101	0.751±0.102	0.689±0.112	0.748±0.079
	DI	0.833±0.097	0.849±0.154	0.838±0.220	0.898±0.069
	FE	0.752±0.188	0.798±0.143	0.835±0.080	0.837±0.079
	HA	0.816±0.153	0.854±0.060	0.839±0.178	0.864±0.084
	SA	0.574±0.111	0.693±0.080	0.652±0.160	0.777±0.020
	SU	0.776±0.085	0.875±0.192	0.854±0.061	0.929±0.066
Bench. 3	AN	0.738±0.163	0.657±0.113	0.662±0.126	0.788±0.058
	DI	0.785±0.226	0.815±0.205	0.789±0.207	0.875±0.028
	FE	0.686±0.135	0.689±0.153	0.662±0.062	0.767±0.011
	HA	0.889±0.120	0.909±0.166	0.841±0.136	0.864±0.071
	SA	0.593±0.220	0.736±0.122	0.659±0.147	0.809±0.044
	SU	0.804±0.122	0.862±0.188	0.915±0.201	0.920±0.065
Proposed	AN	0.792±0.033	0.779±0.096	0.741±0.154	0.929±0.062
	DI	0.842±0.164	0.892±0.264	0.888±0.054	0.937±0.076
	FE	0.755±0.098	0.806±0.095	0.814±0.204	0.903±0.009
	HA	0.824±0.057	0.846±0.147	0.883±0.162	0.857±0.096
	SA	0.574±0.153	0.714±0.160	0.649±0.200	0.892±0.066
	SU	0.869±0.135	0.945±0.107	0.915±0.149	0.958±0.039

Table S4 presents the per-class recognition performance of the benchmark methods and the proposed method using the ResNet-18 backbone under five-fold cross-validation on the JAFFE dataset. Similar to the results obtained with LeNet-5, the proposed feature-fusion strategy consistently improved the recognition performance across most emotion categories.

Compared with the CNN-only baseline (Bench. 1), the proposed method achieved higher F1-scores for all emotion classes. Particularly large improvements were observed for disgust (DI), fear (FE), and happiness (HA), where the F1-score increased from 0.723 ± 0.169 to 0.888 ± 0.054, from 0.723 ± 0.141 to 0.814 ± 0.204, and from 0.769 ± 0.095 to 0.883 ± 0.162, respectively. These improvements indicate that the proposed angular and HOG features effectively complement the deep representations extracted by the ResNet-18 backbone.

Compared with Bench. 2 and Bench. 3, the proposed method also achieved the highest or competitive performance for most emotion categories. In particular, the proposed method consistently provided superior recognition performance for AN, DI, FE, HA, and SU, while maintaining comparable performance for SA. These results demonstrate that the effectiveness of the proposed feature-fusion strategy is preserved even when a stronger CNN backbone is employed.

Overall, the class-wise results further support the findings presented in Table 2, confirming that the proposed handcrafted features improve facial emotion recognition in a balanced manner across different emotion categories without being limited to a specific backbone architecture.

Table S5. Per-class performance of the benchmark methods (Bench. 1–3) and the proposed method using the MobileNetV2 backbone under five-fold cross-validation on the JAFFE dataset.

Setting	Emotion	Macro-Average			ACC
		TPR	PPV	F1	
Bench. 1	AN	0.567±0.224	0.588±0.227	0.575±0.221	0.863±0.071
	DI	0.620±0.247	0.821±0.208	0.674±0.180	0.913±0.045
	FE	0.662±0.147	0.611±0.171	0.630±0.146	0.858±0.075
	HA	0.781±0.235	0.819±0.195	0.774±0.153	0.924±0.055
	SA	0.610±0.156	0.623±0.167	0.594±0.133	0.858±0.061
	SU	0.800±0.183	0.817±0.208	0.794±0.176	0.929±0.065
Bench. 2	AN	0.667±0.118	0.664±0.134	0.657±0.104	0.885±0.041
	DI	0.720±0.284	0.881±0.115	0.763±0.201	0.940±0.045
	FE	0.652±0.147	0.629±0.105	0.632±0.098	0.869±0.037
	HA	0.810±0.211	0.892±0.166	0.830±0.159	0.945±0.051
	SA	0.643±0.186	0.636±0.133	0.627±0.133	0.874±0.037
	SU	0.867±0.217	0.791±0.160	0.820±0.173	0.940±0.053
Bench. 3	AN	0.667±0.118	0.609±0.152	0.630±0.125	0.869±0.052
	DI	0.653±0.265	0.803±0.187	0.684±0.171	0.913±0.040
	FE	0.619±0.150	0.681±0.192	0.631±0.097	0.874±0.037
	HA	0.810±0.211	0.843±0.156	0.803±0.129	0.934±0.041
	SA	0.576±0.258	0.558±0.081	0.542±0.177	0.853±0.024
	SU	0.800±0.217	0.806±0.138	0.793±0.164	0.934±0.050
Proposed	AN	0.633±0.139	0.646±0.099	0.631±0.090	0.880±0.030
	DI	0.760±0.277	0.705±0.184	0.723±0.213	0.913±0.061
	FE	0.657±0.129	0.700±0.192	0.671±0.130	0.885±0.051
	HA	0.876±0.170	0.848±0.156	0.848±0.113	0.945±0.043
	SA	0.581±0.186	0.625±0.072	0.588±0.113	0.869±0.022
	SU	0.800±0.217	0.823±0.177	0.808±0.194	0.939±0.060

Table S5 summarizes the per-class recognition performance of the benchmark methods and the proposed method using the MobileNetV2 backbone under five-fold cross-validation on the JAFFE dataset. Overall, the proposed feature-fusion strategy maintained competitive recognition performance across the six emotion categories while providing noticeable improvements for several representative emotions.

Compared with the CNN-only baseline (Bench. 1), the proposed method achieved higher F1-scores for disgust (DI), fear (FE), and happiness (HA). In particular, the largest improvement was observed for happiness (HA), where the F1-score increased from 0.774 ± 0.153 to 0.848 ± 0.113. Similarly, the F1-scores for disgust (DI) and fear (FE) improved from 0.674 ± 0.180 to 0.723 ± 0.213 and from 0.630 ± 0.146 to 0.671 ± 0.130, respectively. These results indicate that the proposed handcrafted features provide complementary information even when a lightweight CNN architecture is employed.

Compared with Bench. 2 and Bench. 3, the proposed method achieved competitive performance across most emotion categories while maintaining the highest overall recognition performance reported in Table 2. Although the improvement for some individual emotion classes was relatively modest, no substantial degradation was observed. This observation suggests that the proposed feature-fusion strategy provides stable and balanced recognition performance without sacrificing the efficiency advantages of the MobileNetV2 backbone.

Overall, the class-wise results are consistent with the overall performance reported in Table 2, further confirming that the proposed handcrafted features can be effectively integrated with lightweight CNN architectures for facial emotion recognition.

S4.1.2. Statistical Analysis

Table S6 summarizes the paired t-test results comparing the benchmark methods and the proposed method under five-fold cross-validation on the JAFFE dataset. The statistical analysis was conducted to determine whether the observed performance improvements were statistically significant.

For the LeNet-5 backbone, the proposed method achieved statistically significant improvements over the CNN-only baseline (Bench. 1) in terms of accuracy ($p = 0.0080$), TPR ($p = 0.0085$), and F1-score ($p = 0.0117$). These results indicate that the proposed feature-fusion strategy significantly enhances the recognition performance of the lightweight LeNet-5 backbone by providing complementary geometric and texture information.

In contrast, no statistically significant differences were observed between the proposed method and Bench. 2 or Bench. 3 for the LeNet-5 backbone. This observation suggests that both angular features and HOG features individually contribute to recognition performance, while combining the two feature types mainly provides incremental improvements over each individual handcrafted feature.

For the ResNet-18 and MobileNetV2 backbones, most comparisons did not show statistically significant differences ($p > 0.05$). This result is consistent with the stronger feature representation capability of modern CNN architectures, where the additional handcrafted features mainly provide moderate performance improvements rather than large performance gains. Nevertheless, as shown in Table 2, the proposed method consistently achieved the highest or competitive average recognition performance across different backbone networks.

Table S6. Paired *t*-test results comparing the benchmark methods and the proposed method under five-fold cross-validation on the JAFFE dataset.

Backbone	Comparison	Metric	p-value
LeNet-5	Proposed vs Bench. 1	TPR	0.0085
		PPV	0.1489
		F1	0.0117
		ACC	0.0080
	Proposed vs Bench. 2	TPR	0.5580
		PPV	0.7713
		F1	0.5242
		ACC	0.4723
	Proposed vs Bench. 3	TPR	0.9284
		PPV	0.7655
		F1	0.9813
		ACC	0.9913
ResNet-18	Proposed vs Bench. 1	TPR	0.1991
		PPV	0.6217
		F1	0.6121
		ACC	0.1773
	Proposed vs Bench. 2	TPR	0.5460
		PPV	0.2734
		F1	0.5803
		ACC	0.3896
	Proposed vs Bench. 3	TPR	0.4426
		PPV	0.0404
		F1	0.6264
		ACC	0.7988
MobileNetV2	Proposed vs Bench. 1	TPR	0.3275
		PPV	0.7874
		F1	0.3594
		ACC	0.3392
	Proposed vs Bench. 2	TPR	0.4567
		PPV	0.2960
		F1	0.3839
		ACC	0.3702
	Proposed vs Bench. 3	TPR	0.1664
		PPV	0.7465
		F1	0.0999
		ACC	0.1920

S4.2. Results on the CK+ Dataset

S4.2.1. Per-Class Performance

Table S7. Per-class performance of the benchmark methods (Bench. 1–3) and the proposed method using the LeNet-5 backbone under five-fold cross-validation on the CK+ dataset.

Setting	Emotion	Macro-Average			ACC
		TPR	PPV	F1	
Bench. 1	AN	0.356±0.041	0.613±0.187	0.421±0.031	0.874±0.047
	CO	0.567±0.108	0.369±0.053	0.434±0.086	0.924±0.022
	DI	0.639±0.170	0.584±0.092	0.608±0.127	0.856±0.030
	FE	0.280±0.068	0.267±0.053	0.271±0.055	0.887±0.040
	HA	0.810±0.124	0.891±0.084	0.839±0.040	0.936±0.013
	SA	0.633±0.156	0.456±0.029	0.520±0.125	0.893±0.052
	SU	0.833±0.128	0.921±0.074	0.872±0.096	0.939±0.044
Bench. 2	AN	0.533±0.041	0.605±0.085	0.536±0.138	0.884±0.018
	CO	0.633±0.180	0.606±0.129	0.553±0.128	0.948±0.017
	DI	0.744±0.109	0.696±0.136	0.715±0.104	0.893±0.039
	FE	0.360±0.058	0.456±0.040	0.390±0.088	0.921±0.048
	HA	0.911±0.099	0.889±0.092	0.893±0.141	0.954±0.019
	SA	0.540±0.130	0.586±0.062	0.550±0.062	0.920±0.035
	SU	0.952±0.052	0.963±0.056	0.957±0.152	0.979±0.026
Bench. 3	AN	0.556±0.124	0.572±0.044	0.541±0.232	0.890±0.030
	CO	0.633±0.117	0.800±0.174	0.660±0.107	0.963±0.017
	DI	0.761±0.081	0.814±0.156	0.774±0.057	0.917±0.034
	FE	0.360±0.061	0.319±0.092	0.325±0.054	0.899±0.021
	HA	0.928±0.051	0.871±0.077	0.897±0.047	0.954±0.022
	SA	0.607±0.157	0.542±0.099	0.556±0.079	0.917±0.026
	SU	0.928±0.078	0.973±0.037	0.950±0.057	0.976±0.028
Proposed	AN	0.689±0.099	0.813±0.142	0.728±0.134	0.933±0.030
	CO	0.683±0.073	0.750±0.176	0.705±0.148	0.970±0.026
	DI	0.846±0.098	0.840±0.159	0.838±0.040	0.942±0.012
	FE	0.440±0.089	0.499±0.057	0.453±0.066	0.917±0.021
	HA	0.942±0.060	0.887±0.075	0.910±0.028	0.960±0.014
	SA	0.673±0.071	0.652±0.098	0.647±0.065	0.939±0.015
	SU	0.977±0.032	0.966±0.050	0.970±0.029	0.985±0.015

Table S7 presents the per-class recognition performance of the benchmark methods and the proposed method using the LeNet-5 backbone under five-fold cross-validation on the CK+ dataset. The class-wise analysis provides further insight into how the proposed handcrafted features influence the recognition performance of individual emotion categories.

Compared with the CNN-only baseline (Bench. 1), the proposed method consistently improved the F1-score for all seven emotion classes. Particularly large improvements were observed for anger (AN), contempt (CO), disgust (DI), and fear (FE), where the F1-score increased from 0.421 ± 0.031 to 0.728 ± 0.134, from 0.434 ± 0.086 to 0.705 ± 0.148, from 0.608 ± 0.127 to 0.838 ± 0.040, and from 0.271 ± 0.055 to 0.453 ± 0.066, respectively. These results indicate that the proposed angular and HOG features effectively complement CNN-based deep representations, particularly for emotion classes that are relatively difficult to discriminate using deep features alone.

Compared with Bench. 2 and Bench. 3, the proposed method also achieved the highest or competitive performance across all emotion categories. In particular, substantial improvements were observed for disgust (DI), sadness (SA), and surprise (SU), demonstrating

that combining both angular and HOG features provides complementary information beyond using either handcrafted feature individually.

Overall, the class-wise results are consistent with the overall performance reported in Table 5, further confirming that the proposed feature-fusion strategy improves facial emotion recognition in a balanced manner across all emotion categories on the CK+ dataset.

Table S8. Per-class performance of the benchmark methods (Bench. 1–3) and the proposed method using the ResNet-18 backbone under five-fold cross-validation on the CK+ dataset.

Setting	Emotion	Macro-Average			ACC
		TPR	PPV	F1	
Bench. 1	AN	0.548±0.055	0.507±0.182	0.471±0.055	0.853±0.027
	CO	0.484±0.038	0.617±0.105	0.436±0.052	0.936±0.020
	DI	0.766±0.126	0.726±0.168	0.714±0.054	0.928±0.005
	FE	0.202±0.066	0.425±0.028	0.250±0.011	0.959±0.015
	HA	0.958±0.107	0.816±0.011	0.886±0.065	0.930±0.024
	SA	0.381±0.097	0.377±0.043	0.436±0.085	0.915±0.026
	SU	0.978±0.056	0.916±0.148	0.923±0.118	0.951±0.023
Bench. 2	AN	0.473±0.049	0.555±0.114	0.488±0.031	0.905±0.035
	CO	0.429±0.036	0.394±0.046	0.368±0.028	0.911±0.005
	DI	0.783±0.106	0.860±0.101	0.728±0.075	0.899±0.025
	FE	0.105±0.081	0.094±0.051	0.134±0.029	0.940±0.010
	HA	0.879±0.108	0.929±0.084	0.886±0.094	0.920±0.039
	SA	0.390±0.089	0.464±0.084	0.381±0.071	0.931±0.013
	SU	0.946±0.065	0.868±0.138	0.903±0.057	0.924±0.003
Bench. 3	AN	0.506±0.074	0.693±0.099	0.592±0.068	0.931±0.022
	CO	0.459±0.072	0.370±0.085	0.420±0.029	0.929±0.005
	DI	0.709±0.169	0.834±0.088	0.801±0.094	0.935±0.019
	FE	0.148±0.043	0.395±0.037	0.259±0.034	0.946±0.029
	HA	0.903±0.075	0.782±0.086	0.859±0.040	0.930±0.018
	SA	0.381±0.064	0.341±0.030	0.371±0.035	0.891±0.006
	SU	0.957±0.069	0.866±0.067	0.901±0.037	0.953±0.005
Proposed	AN	0.518±0.092	0.584±0.013	0.599±0.027	0.936±0.026
	CO	0.566±0.069	0.617±0.099	0.555±0.087	0.953±0.003
	DI	0.774±0.049	0.820±0.201	0.827±0.091	0.944±0.005
	FE	0.250±0.090	0.340±0.083	0.233±0.046	0.953±0.015
	HA	0.972±0.144	0.934±0.011	0.891±0.130	0.944±0.038
	SA	0.431±0.062	0.490±0.073	0.516±0.055	0.927±0.029
	SU	0.984±0.195	0.876±0.105	0.928±0.051	0.967±0.037

Table S8 presents the per-class recognition performance of the benchmark methods and the proposed method using the ResNet-18 backbone under five-fold cross-validation on the CK+ dataset. Compared with the LeNet-5 backbone, ResNet-18 provides stronger deep feature representations, allowing the effectiveness of the proposed handcrafted features to be evaluated on a more powerful CNN architecture.

Compared with the CNN-only baseline (Bench. 1), the proposed method improved the recognition performance for most emotion categories. In particular, noticeable improvements were observed for anger (AN), contempt (CO), disgust (DI), and sadness (SA), where the F1-score increased from 0.471 ± 0.055 to 0.599 ± 0.027, from 0.436 ± 0.052 to 0.555 ± 0.087, from 0.714 ± 0.054 to 0.827 ± 0.091, and from 0.436 ± 0.085 to 0.516 ± 0.055, respectively. The proposed method also maintained excellent recognition performance for happiness (HA) and surprise (SU), achieving F1-scores of 0.891 ± 0.130 and 0.928 ± 0.051, respectively.

Compared with Bench. 2 and Bench. 3, the proposed method achieved competitive or superior performance for most emotion categories. Although the improvement for fear (FE) remained limited, FE was consistently the most challenging emotion class across all feature settings, indicating that the ambiguity of this expression is largely attributable to the intrinsic characteristics of the dataset rather than the choice of feature representation.

Overall, the class-wise results demonstrate that the proposed handcrafted features continue to provide complementary information even when combined with the stronger ResNet-18 backbone. These findings further support the effectiveness and robustness of the proposed feature-fusion strategy across different CNN architectures.

Table S9. Per-class performance of the benchmark methods (Bench. 1–3) and the proposed method using the MobileNetV2 backbone under five-fold cross-validation on the CK+ dataset.

Setting	Emotion	Macro-Average			ACC
		TPR	PPV	F1	
Bench. 1	AN	0.511±0.102	0.521±0.088	0.498±0.048	0.863±0.037
	CO	0.400±0.081	0.767±0.124	0.488±0.025	0.957±0.007
	DI	0.794±0.106	0.770±0.080	0.775±0.049	0.918±0.017
	FE	0.261±0.040	0.424±0.068	0.267±0.023	0.924±0.015
	HA	0.957±0.039	0.780±0.070	0.859±0.053	0.933±0.027
	SA	0.313±0.051	0.367±0.017	0.308±0.090	0.899±0.030
	SU	0.977±0.032	0.922±0.107	0.945±0.050	0.961±0.029
Bench. 2	AN	0.489±0.022	0.545±0.033	0.504±0.058	0.875±0.030
	CO	0.367±0.080	0.400±0.053	0.368±0.041	0.942±0.007
	DI	0.762±0.110	0.812±0.073	0.781±0.056	0.924±0.018
	FE	0.140±0.029	0.167±0.049	0.150±0.012	0.917±0.009
	HA	0.955±0.068	0.726±0.058	0.824±0.056	0.915±0.025
	SA	0.347±0.042	0.507±0.077	0.367±0.074	0.905±0.025
	SU	0.988±0.026	0.845±0.095	0.909±0.063	0.948±0.038
Bench. 3	AN	0.511±0.127	0.614±0.136	0.545±0.085	0.884±0.023
	CO	0.400±0.081	0.413±0.084	0.394±0.047	0.936±0.012
	DI	0.779±0.098	0.871±0.080	0.819±0.070	0.939±0.022
	FE	0.167±0.061	0.435±0.050	0.217±0.067	0.927±0.013
	HA	0.969±0.069	0.767±0.089	0.854±0.076	0.930±0.035
	SA	0.353±0.099	0.383±0.012	0.360±0.051	0.899±0.021
	SU	0.988±0.026	0.839±0.057	0.906±0.030	0.948±0.018
Proposed	AN	0.467±0.034	0.559±0.052	0.495±0.063	0.918±0.029
	CO	0.567±0.099	0.619±0.130	0.569±0.076	0.951±0.025
	DI	0.814±0.069	0.788±0.030	0.799±0.036	0.927±0.013
	FE	0.279±0.020	0.283±0.091	0.244±0.041	0.921±0.017
	HA	0.969±0.069	0.774±0.099	0.859±0.082	0.933±0.038
	SA	0.387±0.026	0.467±0.083	0.414±0.033	0.905±0.026
	SU	0.988±0.026	0.893±0.050	0.937±0.024	0.966±0.013

Table S9 presents the per-class recognition performance of the benchmark methods and the proposed method using the MobileNetV2 backbone under five-fold cross-validation on the CK+ dataset. Similar to the observations on the JAFFE dataset, the proposed feature-fusion strategy maintained competitive recognition performance while preserving the lightweight characteristics of the MobileNetV2 backbone.

Compared with the CNN-only baseline (Bench. 1), the proposed method improved the F1-score for several representative emotion categories, particularly contempt (CO), disgust (DI), happiness (HA), and sadness (SA). The largest improvements were observed for contempt (CO) and sadness (SA), where the F1-score increased from 0.488 ± 0.025 to 0.569 ± 0.076 and from 0.308 ± 0.190 to 0.414 ± 0.033, respectively. In addition, the proposed

method maintained high recognition performance for happiness (HA) and surprise (SU), which remained among the best-recognized emotion classes.

Compared with Bench. 2 and Bench. 3, the proposed method achieved competitive or superior recognition performance for most emotion categories while maintaining the highest overall performance reported in Table 5. Although the improvement for fear (FE) remained limited, FE consistently exhibited the lowest recognition performance across all feature settings, suggesting that this emotion remains intrinsically difficult to discriminate in the CK+ dataset.

Overall, the class-wise results demonstrate that the proposed handcrafted features can be effectively integrated with lightweight CNN architectures while preserving balanced recognition performance across most emotion categories. These findings further support the practical applicability of the proposed feature-fusion strategy for lightweight facial emotion recognition systems.

Across both JAFFE and CK+, fear consistently exhibited lower recognition performance than the other emotion categories. This suggests that fear remains one of the most challenging facial expressions to recognize because of its subtle facial deformations and similarity to other negative emotions.

S4.2.2. Statistical Analysis

Table S10 summarizes the paired t-test results obtained under five-fold cross-validation on the CK+ dataset. The statistical analysis was conducted to determine whether the performance improvements achieved by the proposed method were statistically significant.

For the LeNet-5 backbone, the proposed method achieved statistically significant improvements over the CNN-only baseline (Bench. 1) for all evaluation metrics ($p < 0.01$). Furthermore, statistically significant improvements were also observed over Bench. 2 in terms of PPV ($p = 0.0448$), F1-score ($p = 0.0458$), and accuracy ($p = 0.0394$). These results indicate that combining both angular and HOG features provides additional discriminative information beyond using angular features alone.

For the ResNet-18 and MobileNetV2 backbones, statistically significant improvements were observed primarily when comparing the proposed method with Bench. 2, particularly for TPR, F1-score, and accuracy. In contrast, no statistically significant differences were observed between the proposed method and Bench. 3. These findings suggest that HOG features contribute substantially to the recognition performance of stronger CNN backbones, whereas the additional angular features provide complementary improvements that are reflected in the overall recognition performance.

Overall, the statistical analysis supports the effectiveness of the proposed feature-fusion strategy across different backbone networks and confirms that integrating handcrafted features with CNN-based deep representations leads to consistent performance improvements.

Table S10. Paired t-test results comparing the benchmark methods and the proposed method under five-fold cross-validation on the CK+ dataset.

Backbone	Comparison	Metric	p-value
LeNet-5	Proposed vs Bench. 1	TPR	0.0037
		PPV	0.0044
		F1	0.0014
		ACC	0.0011
	Proposed vs Bench. 2	TPR	0.0743
		PPV	0.0448
		F1	0.0458
		ACC	0.0394
	Proposed vs Bench. 3	TPR	0.1140
		PPV	0.2297
		F1	0.1343
		ACC	0.0650
ResNet-18	Proposed vs Bench. 1	TPR	0.3446
		PPV	0.5437
		F1	0.6826
		ACC	0.6605
	Proposed vs Bench. 2	TPR	0.0165
		PPV	0.2596
		F1	0.0439
		ACC	0.0277
	Proposed vs Bench. 3	TPR	0.0272
		PPV	0.9721
		F1	0.1829
		ACC	0.3892
MobileNetV2	Proposed vs Bench. 1	TPR	0.3843
		PPV	0.5848
		F1	0.6236
		ACC	0.6213
	Proposed vs Bench. 2	TPR	0.0156
		PPV	0.2811
		F1	0.0449
		ACC	0.0212
	Proposed vs Bench. 3	TPR	0.0196
		PPV	0.9847
		F1	0.1969
		ACC	0.3721

S4.3. Results on the KDEF Dataset

S4.3.1. Per-Class Performance

Table S11. Per-class performance of the benchmark methods (Bench. 1–3) and the proposed method using the LeNet-5 backbone under five-fold cross-validation on the KDEF dataset.

Setting	Emotion	Macro-Average			ACC
		TPR	PPV	F1	
Bench. 1	AN	0.721±0.099	0.755±0.037	0.735±0.057	0.914±0.014
	DI	0.743±0.064	0.749±0.104	0.741±0.059	0.912±0.029
	FE	0.421±0.039	0.506±0.052	0.457±0.027	0.833±0.015
	HA	0.914±0.060	0.906±0.086	0.909±0.064	0.969±0.022
	SA	0.557±0.086	0.534±0.055	0.544±0.066	0.845±0.020
	SU	0.857±0.098	0.764±0.065	0.805±0.057	0.931±0.020
Bench. 2	AN	0.729±0.086	0.815±0.034	0.768±0.059	0.927±0.015
	DI	0.814±0.030	0.810±0.110	0.809±0.060	0.935±0.025
	FE	0.571±0.057	0.717±0.089	0.636±0.069	0.891±0.023
	HA	0.979±0.020	0.940±0.043	0.958±0.026	0.986±0.009
	SA	0.779±0.059	0.693±0.063	0.732±0.053	0.905±0.022
	SU	0.907±0.086	0.817±0.085	0.859±0.078	0.950±0.028
Bench. 3	AN	0.750±0.067	0.829±0.086	0.784±0.048	0.931±0.016
	DI	0.850±0.053	0.862±0.090	0.852±0.034	0.950±0.015
	FE	0.614±0.102	0.719±0.060	0.659±0.066	0.895±0.017
	HA	0.986±0.032	0.940±0.049	0.962±0.039	0.987±0.014
	SA	0.843±0.078	0.777±0.037	0.807±0.045	0.933±0.014
	SU	0.886±0.030	0.824±0.120	0.850±0.068	0.946±0.028
Proposed	AN	0.793±0.059	0.862±0.055	0.825±0.045	0.944±0.014
	DI	0.843±0.060	0.870±0.104	0.851±0.049	0.950±0.020
	FE	0.679±0.119	0.768±0.079	0.714±0.066	0.911±0.017
	HA	0.993±0.016	0.953±0.045	0.972±0.029	0.991±0.010
	SA	0.864±0.081	0.800±0.084	0.827±0.044	0.939±0.017
	SU	0.886±0.047	0.844±0.108	0.861±0.060	0.951±0.024

Table S11 presents the per-class recognition performance of the benchmark methods and the proposed method using the LeNet-5 backbone under five-fold cross-validation on the KDEF dataset. The class-wise analysis further illustrates the contribution of the proposed handcrafted features to the recognition of individual emotion categories.

Compared with the CNN-only baseline (Bench. 1), the proposed method consistently improved the F1-score for all evaluated emotion classes. The largest improvements were observed for fear (FE) and sadness (SA), where the F1-score increased from 0.457 ± 0.027 to 0.714 ± 0.066 and from 0.544 ± 0.066 to 0.827 ± 0.044, respectively. Noticeable improvements were also obtained for anger (AN), where the F1-score increased from 0.735 ± 0.057 to 0.825 ± 0.045. These results indicate that the proposed angular and HOG features effectively enhance the recognition of emotion classes characterized by relatively subtle facial deformations.

Compared with Bench. 2 and Bench. 3, the proposed method also achieved the highest or competitive recognition performance for most emotion categories. In particular, the proposed method achieved the best performance for anger (AN), fear (FE), happiness (HA), sadness (SA), and surprise (SU), while maintaining comparable performance for disgust (DI). These results demonstrate that combining angular and HOG features provides complementary information beyond using either handcrafted feature individually.

Overall, the class-wise results are consistent with the overall performance reported in Table 8, confirming that the proposed feature-fusion strategy improves facial emotion

recognition in a stable and balanced manner across different emotion categories on the KDEF dataset.

Table S12. Per-class performance of the benchmark methods (Bench. 1–3) and the proposed method using the ResNet-18 backbone under five-fold cross-validation on the KDEF dataset.

Setting	Emotion	Macro-Average			ACC
		TPR	PPV	F1	
Bench. 1	AN	0.816±0.069	0.831±0.073	0.811±0.072	0.942±0.020
	DI	0.848±0.038	0.842±0.089	0.833±0.052	0.949±0.037
	FE	0.566±0.141	0.661±0.093	0.615±0.105	0.888±0.021
	HA	0.929±0.037	0.898±0.081	0.922±0.076	0.922±0.027
	SA	0.713±0.097	0.764±0.073	0.744±0.110	0.928±0.029
	SU	0.855±0.047	0.775±0.081	0.796±0.024	0.937±0.020
Bench. 2	AN	0.829±0.092	0.776±0.118	0.802±0.108	0.937±0.019
	DI	0.819±0.034	0.831±0.078	0.813±0.054	0.934±0.033
	FE	0.598±0.114	0.668±0.033	0.570±0.093	0.869±0.050
	HA	0.956±0.046	0.937±0.043	0.944±0.040	0.943±0.018
	SA	0.763±0.029	0.760±0.050	0.769±0.038	0.939±0.020
	SU	0.819±0.035	0.789±0.042	0.806±0.022	0.938±0.038
Bench. 3	AN	0.832±0.043	0.847±0.078	0.830±0.011	0.926±0.031
	DI	0.857±0.066	0.867±0.087	0.839±0.012	0.957±0.020
	FE	0.562±0.114	0.695±0.056	0.661±0.116	0.902±0.031
	HA	0.964±0.045	0.932±0.072	0.954±0.095	0.978±0.016
	SA	0.834±0.024	0.767±0.031	0.774±0.108	0.945±0.022
	SU	0.839±0.049	0.786±0.065	0.786±0.039	0.947±0.013
Proposed	AN	0.825±0.028	0.865±0.084	0.844±0.071	0.945±0.028
	DI	0.908±0.032	0.872±0.073	0.842±0.084	0.953±0.038
	FE	0.609±0.124	0.727±0.094	0.690±0.129	0.896±0.036
	HA	0.985±0.060	0.941±0.043	0.972±0.041	0.982±0.017
	SA	0.846±0.044	0.781±0.032	0.777±0.036	0.949±0.026
	SU	0.868±0.040	0.815±0.087	0.838±0.054	0.951±0.015

Table S12 presents the per-class recognition performance of the benchmark methods and the proposed method using the ResNet-18 backbone under five-fold cross-validation on the KDEF dataset. Similar to the observations on the JAFFE and CK+ datasets, the proposed feature-fusion strategy consistently maintained strong recognition performance across the evaluated emotion categories.

Compared with the CNN-only baseline (Bench. 1), the proposed method improved the F1-score for most emotion classes. In particular, noticeable improvements were observed for anger (AN), fear (FE), happiness (HA), and surprise (SU), where the F1-score increased from 0.811 ± 0.072 to 0.844 ± 0.071, from 0.615 ± 0.105 to 0.690 ± 0.129, from 0.922 ± 0.076 to 0.972 ± 0.041, and from 0.796 ± 0.024 to 0.838 ± 0.054, respectively. These results indicate that the proposed handcrafted features effectively complement the deep representations extracted by the ResNet-18 backbone.

Compared with Bench. 2 and Bench. 3, the proposed method also achieved the highest or competitive performance for most emotion categories. Although the improvements for disgust (DI) and sadness (SA) were relatively modest, no noticeable degradation was observed for any emotion class. These observations suggest that the proposed feature-fusion strategy provides stable and balanced recognition performance even when combined with a stronger CNN backbone.

Overall, the class-wise results further support the findings presented in Table 8, demonstrating that the proposed feature-fusion strategy consistently improves facial emotion

recognition performance across different emotion categories while preserving the advantages of the ResNet-18 backbone.

Table S13. Per-class performance of the benchmark methods (Bench. 1–3) and the proposed method using the MobileNetV2 backbone under five-fold cross-validation on the KDEF dataset.

Setting	Emotion	Macro-Average			ACC
		TPR	PPV	F1	
Bench. 1	AN	0.807±0.093	0.818±0.069	0.809±0.059	0.937±0.019
	DI	0.843±0.048	0.840±0.118	0.837±0.055	0.944±0.023
	FE	0.579±0.148	0.671±0.081	0.611±0.110	0.881±0.024
	HA	0.936±0.030	0.896±0.097	0.912±0.048	0.969±0.020
	SA	0.729±0.112	0.752±0.074	0.737±0.080	0.914±0.022
	SU	0.829±0.047	0.771±0.073	0.796±0.028	0.929±0.013
Bench. 2	AN	0.821±0.088	0.772±0.093	0.794±0.079	0.929±0.028
	DI	0.807±0.048	0.800±0.088	0.803±0.064	0.933±0.024
	FE	0.536±0.104	0.672±0.060	0.594±0.087	0.880±0.018
	HA	0.957±0.030	0.921±0.049	0.938±0.022	0.979±0.008
	SA	0.757±0.059	0.742±0.043	0.749±0.043	0.916±0.014
	SU	0.814±0.059	0.773±0.079	0.793±0.067	0.929±0.025
Bench. 3	AN	0.814±0.047	0.846±0.085	0.827±0.045	0.943±0.017
	DI	0.850±0.039	0.862±0.089	0.855±0.061	0.951±0.022
	FE	0.550±0.097	0.688±0.080	0.609±0.085	0.883±0.024
	HA	0.971±0.030	0.927±0.055	0.948±0.039	0.982±0.014
	SA	0.814±0.053	0.738±0.033	0.773±0.008	0.920±0.003
	SU	0.829±0.030	0.776±0.084	0.799±0.049	0.930±0.020
Proposed	AN	0.821±0.067	0.837±0.100	0.826±0.062	0.942±0.022
	DI	0.843±0.070	0.837±0.114	0.839±0.087	0.945±0.030
	FE	0.564±0.148	0.714±0.114	0.624±0.126	0.889±0.032
	HA	0.971±0.030	0.935±0.061	0.952±0.034	0.983±0.012
	SA	0.814±0.077	0.761±0.039	0.785±0.046	0.926±0.014
	SU	0.857±0.036	0.796±0.077	0.823±0.044	0.938±0.018

Table S13 presents the per-class recognition performance of the benchmark methods and the proposed method using the MobileNetV2 backbone under five-fold cross-validation on the KDEF dataset. Consistent with the observations on the JAFFE and CK+ datasets, the proposed feature-fusion strategy maintained stable recognition performance while preserving the lightweight characteristics of the MobileNetV2 backbone.

Compared with the CNN-only baseline (Bench. 1), the proposed method improved the F1-score for several representative emotion categories, particularly fear (FE), happiness (HA), sadness (SA), and surprise (SU). The largest improvement was observed for sadness (SA), where the F1-score increased from 0.737 ± 0.080 to 0.785 ± 0.046. Improvements were also observed for fear (FE), happiness (HA), and surprise (SU), demonstrating that the proposed handcrafted features effectively complement the deep representations extracted by the MobileNetV2 backbone.

Compared with Bench. 2 and Bench. 3, the proposed method achieved competitive or superior recognition performance for most emotion categories. Although the improvement for anger (AN) and disgust (DI) was relatively modest, no noticeable performance degradation was observed. These observations indicate that the proposed feature-fusion strategy provides stable and balanced recognition performance while maintaining the computational advantages of the lightweight MobileNetV2 architecture.

Overall, the class-wise results further support the findings presented in Table 8, confirming that the proposed feature-fusion strategy can be effectively integrated with

lightweight CNN architectures while consistently improving facial emotion recognition performance across different emotion categories.

S4.3.2. Statistical Analysis

Table S14. Paired *t*-test results comparing the benchmark methods and the proposed method under five-fold cross-validation on the CK+ dataset.

Backbone	Comparison	Metric	p-value
LeNet-5	Proposed vs Bench. 1	TPR	<0.0001
		PPV	<0.0001
		F1	<0.0001
		ACC	<0.0001
	Proposed vs Bench. 2	TPR	0.0029
		PPV	0.0051
		F1	0.0038
		ACC	0.0029
	Proposed vs Bench. 3	TPR	0.0008
		PPV	0.0030
		F1	0.0028
		ACC	0.0008
ResNet-18	Proposed vs Bench. 1	TPR	0.2105
		PPV	0.2611
		F1	0.2321
		ACC	0.2105
	Proposed vs Bench. 2	TPR	0.0503
		PPV	0.0393
		F1	0.0576
		ACC	0.0503
	Proposed vs Bench. 3	TPR	0.5650
		PPV	0.5871
		F1	0.5991
		ACC	0.5650
MobileNetV2	Proposed vs Bench. 1	TPR	0.2150
		PPV	0.2993
		F1	0.2662
		ACC	0.2150
	Proposed vs Bench. 2	TPR	0.0491
		PPV	0.0296
		F1	0.0536
		ACC	0.0491
	Proposed vs Bench. 3	TPR	0.5291
		PPV	0.5433
		F1	0.5891
		ACC	0.5291

Table S14 summarizes the paired *t*-test results obtained under five-fold cross-validation on the KDEF dataset. The statistical analysis was performed to determine whether the performance improvements achieved by the proposed method were statistically significant.

For the LeNet-5 backbone, the proposed method achieved statistically significant improvements over all benchmark settings. Compared with Bench. 1, statistically significant improvements were observed for all evaluation metrics ($p < 0.0001$). Furthermore, the proposed method also significantly outperformed Bench. 2 ($p < 0.01$ for all metrics) and Bench. 3 ($p < 0.01$ for all metrics). These results provide strong statistical evidence

that combining both angular and HOG features consistently improves the recognition performance of the lightweight LeNet-5 backbone.

For the ResNet-18 and MobileNetV2 backbones, statistically significant improvements were primarily observed when comparing the proposed method with Bench. 2. In particular, the proposed method achieved statistically significant improvements in PPV ($p = 0.0393$) for ResNet-18 and PPV ($p = 0.0296$) for MobileNetV2, whereas the remaining comparisons did not reach the conventional significance level ($p > 0.05$). These findings indicate that stronger CNN backbones already provide highly discriminative deep representations, thereby reducing the magnitude of the additional improvement introduced by handcrafted features.

Overall, the statistical analysis demonstrates that the proposed feature-fusion strategy provides the greatest statistical benefit for lightweight CNN architectures while maintaining competitive performance improvements for stronger backbone networks.

S5. Results under Leave-One-Subject-Out Cross-Validation

S5.1. Statistical Analysis on the JAFFE Dataset

Table S15 summarizes the paired t-test results obtained under LOSO cross-validation on the JAFFE dataset. The statistical analysis was conducted to evaluate whether the observed improvements under the subject-independent evaluation protocol were statistically significant.

For the LeNet-5 backbone, the proposed method achieved statistically significant improvements over the CNN-only baseline (Bench. 1) for all evaluation metrics ($p < 0.01$). Furthermore, statistically significant improvements were also observed over Bench. 3 in terms of TPR ($p = 0.0386$), F1-score ($p = 0.0249$), and accuracy ($p = 0.0432$). Compared with Bench. 2, the proposed method also achieved a statistically significant improvement in F1-score ($p = 0.0457$). These results demonstrate that combining angular and HOG features significantly improves the subject-independent recognition capability of the lightweight LeNet-5 backbone.

For the ResNet-18 and MobileNetV2 backbones, no statistically significant differences were observed between the proposed method and the benchmark settings ($p > 0.05$). Although the proposed method consistently achieved higher average recognition performance, the statistical analysis suggests that the stronger deep feature representations provided by these backbone networks reduce the magnitude of the additional improvement introduced by handcrafted features under the LOSO evaluation protocol.

Overall, the statistical analysis indicates that the proposed feature-fusion strategy provides statistically significant improvements for lightweight CNN architectures while maintaining competitive subject-independent recognition performance for stronger backbone networks.

Table S15. Paired *t*-test results comparing the benchmark methods and the proposed method under leave-one-subject-out cross-validation on the JAFFE dataset.

Backbone	Comparison	Metric	p-value
LeNet-5	Proposed vs Bench. 1	TPR	0.0009
		PPV	0.0019
		F1	0.0012
		ACC	0.0009
	Proposed vs Bench. 2	TPR	0.0998
		PPV	0.1149
		F1	0.0457
		ACC	0.0908
	Proposed vs Bench. 3	TPR	0.0386
		PPV	0.0903
		F1	0.0249
		ACC	0.0432
ResNet-18	Proposed vs Bench. 1	TPR	0.9776
		PPV	0.8968
		F1	0.7833
		ACC	0.9105
	Proposed vs Bench. 2	TPR	0.7336
		PPV	0.6324
		F1	0.5631
		ACC	0.3179
	Proposed vs Bench. 3	TPR	0.1295
		PPV	0.8703
		F1	0.5369
		ACC	0.1279
MobileNetV2	Proposed vs Bench. 1	TPR	0.9777
		PPV	0.8904
		F1	0.8009
		ACC	0.9105
	Proposed vs Bench. 2	TPR	0.3492
		PPV	0.6830
		F1	0.5723
		ACC	0.3306
	Proposed vs Bench. 3	TPR	0.1199
		PPV	0.7948
		F1	0.5674
		ACC	0.1146

S5.2. Statistical Analysis on the KDEF Dataset

Table S16 summarizes the paired *t*-test results obtained under leave-one-subject-out (LOSO) cross-validation on the KDEF dataset. The statistical analysis was performed to determine whether the performance improvements achieved by the proposed method under the subject-independent evaluation protocol were statistically significant.

For the LeNet-5 backbone, the proposed method achieved statistically significant improvements over the CNN-only baseline (Bench. 1) for all evaluation metrics ($p < 0.001$). In addition, statistically significant improvements were observed over Bench. 2 in terms of PPV ($p = 0.0038$) and F1-score ($p = 0.0280$), while the proposed method significantly outperformed Bench. 3 for all evaluation metrics ($p < 0.01$). These results provide strong statistical evidence that combining both angular and HOG features substantially enhances the subject-independent recognition capability of the lightweight LeNet-5 backbone.

For the ResNet-18 and MobileNetV2 backbones, no statistically significant differences were observed between the proposed method and the benchmark settings ($p > 0.05$).

Although the proposed method consistently achieved higher average recognition performance, the statistical analysis suggests that the stronger deep feature representations learned by these backbone networks reduce the magnitude of the additional improvement introduced by handcrafted features under LOSO evaluation.

Overall, the statistical analysis further supports the effectiveness of the proposed feature-fusion strategy for subject-independent facial emotion recognition, with the most pronounced statistical benefits consistently observed for the lightweight LeNet-5 backbone.

Table S16. Paired *t*-test results comparing the benchmark methods and the proposed method under leave-one-subject-out cross-validation on the KDEF dataset.

Backbone	Comparison	Metric	p-value
LeNet-5	Proposed vs Bench. 1	TPR	<0.001
		PPV	<0.001
		F1	<0.001
		ACC	<0.001
	Proposed vs Bench. 2	TPR	0.1247
		PPV	0.0038
		F1	0.0280
		ACC	0.1247
	Proposed vs Bench. 3	TPR	0.0037
		PPV	0.0096
		F1	0.0051
		ACC	0.0037
ResNet-18	Proposed vs Bench. 1	TPR	0.6657
		PPV	0.3054
		F1	0.5558
		ACC	0.6657
	Proposed vs Bench. 2	TPR	0.2893
		PPV	0.4607
		F1	0.2259
		ACC	0.2893
	Proposed vs Bench. 3	TPR	0.1123
		PPV	0.6209
		F1	0.1815
		ACC	0.1123
MobileNetV2	Proposed vs Bench. 1	TPR	0.6385
		PPV	0.2547
		F1	0.4703
		ACC	0.6385
	Proposed vs Bench. 2	TPR	0.2346
		PPV	0.4156
		F1	0.2075
		ACC	0.2346
	Proposed vs Bench. 3	TPR	0.0628
		PPV	0.6072
		F1	0.1503
		ACC	0.0628

S6. Results on the Official RAF-DB Train/Test Split

Table S17. Per-class performance of the benchmark methods (Bench. 1–3) and the proposed method using the LeNet-5 backbone on the RAF-DB dataset.

Setting	Emotion	Macro-Average			ACC
		TPR	PPV	F1	
Bench. 1	AN	0.6915	0.8025	0.7429	0.9654
	DI	0.4965	0.5621	0.5273	0.9345
	FE	0.3600	0.8438	0.5047	0.9796
	HA	0.9151	0.9125	0.9138	0.9155
	SA	0.7880	0.7484	0.7677	0.9030
	SU	0.8517	0.7258	0.7838	0.9378
Bench. 2	AN	0.5432	0.5535	0.5483	0.9393
	DI	0.3312	0.3985	0.3618	0.9217
	FE	0.1622	0.8571	0.2727	0.9732
	HA	0.8751	0.8404	0.8574	0.8555
	SA	0.6548	0.5962	0.6241	0.8421
	SU	0.6930	0.7059	0.6994	0.9179
Bench. 3	AN	0.5556	0.5455	0.5505	0.9384
	DI	0.2000	0.5161	0.2883	0.9338
	FE	0.1757	0.8667	0.2921	0.9736
	HA	0.8743	0.8229	0.8478	0.8442
	SA	0.6423	0.5825	0.6109	0.8363
	SU	0.7234	0.6611	0.6909	0.9108
Proposed	AN	0.5926	0.5963	0.5944	0.9451
	DI	0.4062	0.4362	0.4207	0.9250
	FE	0.3378	0.7143	0.4587	0.9753
	HA	0.8886	0.8431	0.8652	0.8626
	SA	0.6234	0.6478	0.6354	0.8568
	SU	0.7143	0.7036	0.7089	0.9192

Table S17 presents the per-class recognition performance of the benchmark methods and the proposed method using the LeNet-5 backbone on the official RAF-DB train/test split. Compared with the controlled datasets, the per-class performance on RAF-DB exhibited greater variability because of the increased complexity of in-the-wild facial expression recognition.

Although the proposed method did not achieve the highest F1-score for every individual emotion category, it consistently maintained competitive recognition performance across all evaluated classes. In particular, noticeable improvements over Bench. 2 and Bench. 3 were observed for disgust (DI), fear (FE), sadness (SA), and surprise (SU), indicating that the proposed angular and HOG features provide complementary information under more challenging real-world conditions.

For several emotion categories, such as anger (AN) and happiness (HA), the CNN-only baseline (Bench. 1) achieved slightly higher class-wise F1-scores. Nevertheless, as shown in Table 15, the proposed method achieved the highest overall recognition performance across all evaluation metrics, suggesting that the proposed feature-fusion strategy provides a more balanced representation across different facial expression categories rather than optimizing only a small subset of emotions.

Overall, the class-wise results demonstrate that the proposed feature-fusion strategy remains effective under realistic in-the-wild conditions while maintaining competitive recognition performance across diverse facial expression categories.

Table S18. Per-class performance of the benchmark methods (Bench. 1–3) and the proposed method using the ResNet-18 backbone on the RAF-DB dataset.

Setting	Emotion	Macro-Average			ACC
		TPR	PPV	F1	
Bench. 1	AN	0.3439	0.4833	0.4019	0.9119
	DI	0.1697	0.3702	0.2327	0.8984
	FE	0.2794	0.5992	0.3811	0.9129
	HA	0.8377	0.7359	0.7835	0.7666
	SA	0.5938	0.5756	0.5846	0.8150
	SU	0.6230	0.6552	0.6387	0.8555
Bench. 2	AN	0.4709	0.4905	0.4805	0.9187
	DI	0.2703	0.4239	0.3301	0.9026
	FE	0.2143	0.5434	0.3074	0.9299
	HA	0.8233	0.7603	0.7905	0.7791
	SA	0.6007	0.5809	0.5906	0.8159
	SU	0.6526	0.6422	0.6474	0.8829
Bench. 3	AN	0.4265	0.4897	0.4559	0.9217
	DI	0.2837	0.4708	0.3541	0.9087
	FE	0.2912	0.4815	0.3629	0.9318
	HA	0.8351	0.8078	0.8212	0.8040
	SA	0.6132	0.5788	0.5955	0.8177
	SU	0.6472	0.6381	0.6426	0.8901
Proposed	AN	0.5326	0.5353	0.5339	0.9221
	DI	0.3137	0.5068	0.3875	0.9106
	FE	0.3173	0.5162	0.393	0.9257
	HA	0.8407	0.8108	0.8255	0.8391
	SA	0.6541	0.6093	0.6309	0.8263
	SU	0.6581	0.6400	0.6489	0.9019

Table S18 presents the per-class recognition performance of the benchmark methods and the proposed method using the ResNet-18 backbone on the official RAF-DB train/test split. Compared with the controlled datasets, the RAF-DB dataset exhibits considerably larger intra-class variability, making per-class facial emotion recognition substantially more challenging.

Compared with the CNN-only baseline (Bench. 1), the proposed method improved the F1-score for all evaluated emotion categories. Particularly noticeable improvements were observed for anger (AN), disgust (DI), happiness (HA), and sadness (SA), where the F1-score increased from 0.4019 to 0.5339, from 0.2327 to 0.3875, from 0.7835 to 0.8255, and from 0.5846 to 0.6309, respectively. These results indicate that the proposed angular and HOG features continue to provide complementary information even when combined with the stronger ResNet-18 backbone under realistic facial emotion recognition conditions.

Compared with Bench. 2 and Bench. 3, the proposed method also achieved competitive or superior recognition performance for most emotion categories while maintaining the highest overall recognition performance reported in Table 15. Although the improvements for fear (FE) and surprise (SU) were relatively modest, no substantial performance degradation was observed for any emotion category.

Overall, the class-wise results further demonstrate that the proposed feature-fusion strategy provides stable and balanced recognition performance across diverse emotion categories, even under the challenging in-the-wild conditions represented by the RAF-DB dataset.

Table S19. Per-class performance of the benchmark methods (Bench. 1–3) and the proposed method using the MobileNetV2 backbone on the RAF-DB dataset.

Setting	Emotion	Macro-Average			ACC
		TPR	PPV	F1	
Bench. 1	AN	0.3827	0.5167	0.4397	0.9338
	DI	0.1938	0.4133	0.2638	0.9276
	FE	0.3108	0.6216	0.4144	0.9728
	HA	0.8565	0.7447	0.7967	0.7831
	SA	0.6004	0.5881	0.5942	0.8358
	SU	0.5866	0.6328	0.6088	0.8961
Bench. 2	AN	0.5062	0.5093	0.5077	0.9334
	DI	0.2938	0.4796	0.3643	0.9313
	FE	0.2703	0.5556	0.3636	0.9707
	HA	0.8574	0.8096	0.8328	0.8291
	SA	0.6402	0.6024	0.6207	0.8434
	SU	0.6809	0.6788	0.6798	0.9116
Bench. 3	AN	0.4568	0.5103	0.4821	0.9334
	DI	0.2938	0.5000	0.3701	0.9330
	FE	0.3108	0.4423	0.3651	0.9665
	HA	0.8447	0.8225	0.8335	0.8325
	SA	0.6715	0.5879	0.6270	0.8400
	SU	0.6565	0.6467	0.6516	0.9033
Proposed	AN	0.5370	0.5506	0.5438	0.9389
	DI	0.3312	0.5146	0.4030	0.9343
	FE	0.3378	0.5319	0.4132	0.9703
	HA	0.8709	0.8350	0.8525	0.8505
	SA	0.6904	0.6310	0.6593	0.8572
	SU	0.6626	0.6791	0.6708	0.9104

Table S19 presents the per-class recognition performance of the benchmark methods and the proposed method using the MobileNetV2 backbone on the official RAF-DB train/test split. Similar to the results obtained with the LeNet-5 and ResNet-18 backbones, the proposed feature-fusion strategy maintained competitive recognition performance under the challenging in-the-wild evaluation setting while preserving the efficiency advantages of the lightweight MobileNetV2 architecture.

Compared with the CNN-only baseline (Bench. 1), the proposed method improved the F1-score for most emotion categories. Particularly noticeable improvements were observed for anger (AN), disgust (DI), happiness (HA), sadness (SA), and surprise (SU), where the F1-score increased from 0.4397 to 0.5438, from 0.2638 to 0.4030, from 0.7967 to 0.8525, from 0.5942 to 0.6593, and from 0.6088 to 0.6708, respectively. Although the improvement for fear (FE) was marginal, the proposed method maintained competitive recognition performance for this challenging emotion category.

Compared with Bench. 2 and Bench. 3, the proposed method also achieved competitive or superior recognition performance for most emotion categories. These observations indicate that the proposed angular and HOG features effectively complement the deep representations learned by the lightweight MobileNetV2 backbone, leading to more balanced recognition performance under realistic facial emotion recognition conditions.

Overall, the class-wise results further support the findings presented in Table 15, demonstrating that the proposed feature-fusion strategy can be effectively integrated with lightweight CNN architectures while maintaining robust recognition performance on the challenging RAF-DB dataset.

S7. Results on the Official AffectNet Train/Test Split

Table S20. Per-class performance of the benchmark methods (Bench. 1–3) and the proposed method using the LeNet-5 backbone on the AffectNet dataset.

Setting	Emotion	Macro-Average			ACC
		TPR	PPV	F1	
Bench. 1	AN	0.2888	0.4260	0.3442	0.8322
	CO	0.4971	0.3667	0.4220	0.8406
	DI	0.1065	0.3981	0.1680	0.8809
	FE	0.1212	0.4013	0.1862	0.8428
	HA	0.9178	0.8408	0.8776	0.9527
	SA	0.5821	0.3153	0.4090	0.7654
	SU	0.4343	0.3088	0.3609	0.7773
Bench. 2	AN	0.3121	0.4357	0.3637	0.8334
	CO	0.4979	0.4963	0.4971	0.8821
	DI	0.2581	0.4226	0.3205	0.8764
	FE	0.2975	0.4698	0.3643	0.8459
	HA	0.9428	0.8225	0.8785	0.9518
	SA	0.6366	0.3618	0.4614	0.7927
	SU	0.4243	0.4011	0.4124	0.8249
Bench. 3	AN	0.2327	0.4749	0.3123	0.8437
	CO	0.5399	0.4660	0.5002	0.8737
	DI	0.2871	0.4202	0.3411	0.8748
	FE	0.1542	0.5805	0.2437	0.8580
	HA	0.9376	0.8077	0.8678	0.9472
	SA	0.6655	0.3114	0.4243	0.7481
	SU	0.4057	0.3661	0.3849	0.8122
Proposed	AN	0.3052	0.4722	0.3707	0.8420
	CO	0.5349	0.5233	0.5291	0.8885
	DI	0.3842	0.4199	0.4012	0.8706
	FE	0.3001	0.4884	0.3717	0.8495
	HA	0.9246	0.8580	0.8901	0.9578
	SA	0.6766	0.3933	0.4975	0.8094
	SU	0.4329	0.4228	0.4278	0.8323

Table S20 presents the per-class recognition performance of the benchmark methods and the proposed method using the LeNet-5 backbone on the official AffectNet train/test split. The class-wise analysis provides further insight into the effectiveness of the proposed feature-fusion strategy under a highly challenging large-scale in-the-wild evaluation setting.

Compared with the CNN-only baseline (Bench. 1), the proposed method improved the F1-score for all evaluated emotion categories. Particularly large improvements were observed for contempt (CO), disgust (DI), fear (FE), and sadness (SA), where the F1-score increased from 0.4220 to 0.5291, from 0.1680 to 0.4012, from 0.1862 to 0.3717, and from 0.4090 to 0.4975, respectively. These results indicate that the proposed angular and HOG features effectively capture complementary geometric and local texture information even under substantial variations in facial appearance and imaging conditions.

Compared with Bench. 2 and Bench. 3, the proposed method also achieved the highest or competitive recognition performance across all emotion categories. In particular, the proposed method consistently maintained high recognition performance for happiness (HA) while substantially improving several challenging emotion classes, resulting in a more balanced class-wise recognition performance.

Overall, the class-wise results further support the findings presented in Table 18, demonstrating that the proposed feature-fusion strategy generalizes effectively to large-scale in-the-wild facial emotion recognition while maintaining robust performance across diverse emotion categories.

Table S21. Per-class performance of the benchmark methods (Bench. 1–3) and the proposed method using the ResNet-18 backbone on the AffectNet dataset.

Setting	Emotion	Macro-Average			ACC
		TPR	PPV	F1	
Bench. 1	AN	0.2640	0.4625	0.3361	0.8073
	CO	0.4395	0.3364	0.3811	0.8242
	DI	0.1041	0.3884	0.1642	0.8316
	FE	0.1309	0.4783	0.2055	0.8227
	HA	0.9120	0.8369	0.8728	0.9568
	SA	0.5615	0.3711	0.4469	0.7201
	SU	0.4381	0.3029	0.3582	0.7652
Bench. 2	AN	0.2721	0.3966	0.3228	0.8277
	CO	0.5204	0.4532	0.4845	0.8745
	DI	0.2944	0.4152	0.3445	0.8814
	FE	0.2621	0.4597	0.3339	0.8972
	HA	0.9118	0.8339	0.8711	0.9201
	SA	0.5543	0.3567	0.4341	0.7407
	SU	0.4252	0.3992	0.4118	0.7986
Bench. 3	AN	0.2706	0.5057	0.3526	0.7903
	CO	0.5474	0.4589	0.4993	0.8791
	DI	0.3030	0.4571	0.3644	0.8568
	FE	0.1449	0.5812	0.2320	0.8382
	HA	0.9256	0.7906	0.8528	0.9151
	SA	0.6733	0.2874	0.4028	0.7454
	SU	0.4367	0.3493	0.3881	0.7508
Proposed	AN	0.3916	0.4856	0.4336	0.8239
	CO	0.5681	0.5534	0.5607	0.8734
	DI	0.3944	0.4677	0.4279	0.8748
	FE	0.3892	0.4871	0.4327	0.9149
	HA	0.9350	0.8497	0.8903	0.9595
	SA	0.6987	0.4310	0.5331	0.8370
	SU	0.5663	0.4777	0.5182	0.8273

Table S21 presents the per-class recognition performance of the benchmark methods and the proposed method using the ResNet-18 backbone on the official AffectNet train/test split. Similar to the observations obtained with the LeNet-5 backbone, the proposed feature-fusion strategy consistently improved the recognition performance across the evaluated emotion categories despite the substantial complexity of the AffectNet dataset.

Compared with the CNN-only baseline (Bench. 1), the proposed method improved the F1-score for all evaluated emotion categories. Particularly large improvements were observed for contempt (CO), disgust (DI), fear (FE), surprise (SU), and sadness (SA), where the F1-score increased from 0.3811 to 0.5607, from 0.1642 to 0.4279, from 0.2055 to 0.4327, from 0.3582 to 0.5182, and from 0.4469 to 0.5331, respectively. These substantial improvements demonstrate that the proposed handcrafted angular and HOG features effectively complement the stronger deep representations learned by the ResNet-18 backbone.

Compared with Bench. 2 and Bench. 3, the proposed method also achieved the highest or competitive recognition performance across nearly all emotion categories. In particular, the proposed method maintained high recognition performance for happiness (HA) while

substantially improving several challenging negative emotion categories, resulting in more balanced class-wise recognition performance under large-scale in-the-wild conditions.

Overall, the class-wise results further support the findings presented in Table 18, confirming that the proposed feature-fusion strategy remains effective when integrated with stronger CNN backbones on large-scale real-world facial emotion recognition datasets.

Table S22. Per-class performance of the benchmark methods (Bench. 1–3) and the proposed method using the MobileNetV2 backbone on the AffectNet dataset.

Setting	Emotion	Macro-Average			ACC
		TPR	PPV	F1	
Bench. 1	AN	0.2270	0.4800	0.3082	0.8446
	CO	0.5103	0.4190	0.4602	0.8599
	DI	0.1090	0.4103	0.1723	0.8817
	FE	0.3448	0.4801	0.4014	0.8474
	HA	0.8824	0.6948	0.7774	0.9066
	SA	0.6966	0.3078	0.4270	0.7393
	SU	0.3094	0.4551	0.3684	0.8463
Bench. 2	AN	0.2945	0.4689	0.3617	0.8415
	CO	0.5168	0.5040	0.5103	0.8839
	DI	0.2215	0.3641	0.2754	0.8684
	FE	0.3221	0.5087	0.3944	0.8532
	HA	0.8933	0.7263	0.8012	0.9181
	SA	0.5959	0.3691	0.4558	0.8016
	SU	0.5027	0.4306	0.4638	0.8317
Bench. 3	AN	0.3298	0.4373	0.3760	0.8330
	CO	0.5012	0.4892	0.4951	0.8804
	DI	0.2717	0.3789	0.3165	0.8675
	FE	0.3046	0.4891	0.3754	0.8496
	HA	0.9048	0.7018	0.7905	0.9113
	SA	0.5448	0.3926	0.4564	0.8190
	SU	0.4768	0.4320	0.4533	0.8334
Proposed	AN	0.2907	0.4812	0.3624	0.8440
	CO	0.5275	0.4853	0.5055	0.8792
	DI	0.2632	0.4221	0.3242	0.8761
	FE	0.3299	0.4880	0.3937	0.8492
	HA	0.8975	0.7442	0.8137	0.9240
	SA	0.5972	0.3827	0.4665	0.8095
	SU	0.4947	0.4231	0.4561	0.8291

Table S22 presents the per-class recognition performance of the benchmark methods and the proposed method using the MobileNetV2 backbone on the official AffectNet train/test split. Similar to the observations obtained with the LeNet-5 and ResNet-18 backbones, the proposed feature-fusion strategy maintained competitive recognition performance under the highly challenging large-scale in-the-wild evaluation setting while preserving the efficiency advantages of the lightweight MobileNetV2 architecture.

Compared with the CNN-only baseline (Bench. 1), the proposed method improved the F1-score for most evaluated emotion categories. Particularly noticeable improvements were observed for anger (AN), contempt (CO), disgust (DI), happiness (HA), and sadness (SA), where the F1-score increased from 0.3082 to 0.3624, from 0.4602 to 0.5055, from 0.1723 to 0.3242, from 0.7774 to 0.8137, and from 0.4270 to 0.4665, respectively. Although the improvements for fear (FE) and surprise (SU) were relatively limited, the proposed method maintained competitive recognition performance for these challenging emotion categories.

Compared with Bench. 2 and Bench. 3, the proposed method also achieved competitive or superior recognition performance for most emotion categories. These results indicate that the proposed angular and HOG features effectively complement the deep representations learned by the lightweight MobileNetV2 backbone, leading to more balanced recognition performance under highly diverse real-world conditions.

Overall, the class-wise results further support the findings presented in Table 18, demonstrating that the proposed feature-fusion strategy can be effectively integrated with lightweight CNN architectures while maintaining robust and balanced recognition performance on the large-scale AffectNet dataset.

Disclaimer/Publisher's Note: The statements, opinions and data contained in all publications are solely those of the individual author(s) and contributor(s) and not of MDPI and/or the editor(s). MDPI and/or the editor(s) disclaim responsibility for any injury to people or property resulting from any ideas, methods, instructions or products referred to in the content.
